# The role of GRHL2 and epigenetic remodeling in epithelial–mesenchymal plasticity in ovarian cancer cells

**DOI:** 10.1038/s42003-019-0506-3

**Published:** 2019-07-24

**Authors:** Vin Yee Chung, Tuan Zea Tan, Jieru Ye, Rui-Lan Huang, Hung-Cheng Lai, Dennis Kappei, Heike Wollmann, Ernesto Guccione, Ruby Yun-Ju Huang

**Affiliations:** 1 Cancer Science Institute of Singapore, National University of Singapore, Singapore, 117599 Singapore; 2 Department of Obstetrics and Gynecology, Shuang Ho Hospital, Taipei Medical University, 11031 Taipei, Taiwan; 3 Department of Biochemistry, Yong Loo Lin School of Medicine, National University of Singapore, Singapore, 117596 Singapore; 4 Institute of Molecular and Cell Biology, A*STAR, Singapore, 138673 Singapore; 5 School of Medicine, College of Medicine, National Taiwan University, 10051 Taipei, Taiwan

**Keywords:** Epigenetics, Ovarian cancer, Differentiation

## Abstract

Cancer cells exhibit phenotypic plasticity during epithelial–mesenchymal transition (EMT) and mesenchymal–epithelial transition (MET) involving intermediate states. To study genome-wide epigenetic remodeling associated with EMT plasticity, we integrate the analyses of DNA methylation, ChIP-sequencing of five histone marks (H3K4me1, H3K4me3, H3K27Ac, H3K27me3 and H3K9me3) and transcriptome profiling performed on ovarian cancer cells with different epithelial/mesenchymal states and on a knockdown model of EMT suppressor Grainyhead-like 2 (GRHL2). We have identified differentially methylated CpG sites associated with EMT, found at promoters of epithelial genes and GRHL2 binding sites. GRHL2 knockdown results in CpG methylation gain and nucleosomal remodeling (reduction in permissive marks H3K4me3 and H3K27ac; elevated repressive mark H3K27me3), resembling the changes observed across progressive EMT states. Epigenetic-modifying agents such as 5-azacitidine, GSK126 and mocetinostat further reveal cell state-dependent plasticity upon GRHL2 overexpression. Overall, we demonstrate that epithelial genes are subject to epigenetic control during intermediate phases of EMT/MET involving GRHL2.

## Abstract (web summary)

Vin Yee Chung et al. identify differentially methylated CpG sites in the promoters of epithelial genes and GRHL2 binding sites in ovarian cancer cells during the epithelial–mesenchymal transition. They find that GRHL2 knockdown or overexpression affects methylation, suggesting a role for this transcription factor in epigenetic remodeling.

## Introduction

Despite being two distinct cell states, epithelial and mesenchymal states are capable of trans-differentiating into each other through epithelial–mesenchymal transition (EMT) and its reverse, mesenchymal–epithelial transition (MET)^1^. In response to EMT-inducing stimuli such as transforming growth factor-β (TGF-β), the typical EMT is hallmarked by loss of epithelial proteins (E-cadherin, cytokeratins, etc.) and concomitant gains of mesenchymal-specific proteins (vimentin, N-cadherin, fibronectin, etc.), which lead to the weakening of cell–cell adhesions and strengthening of cell–matrix invasion. With the observations of intermediate/hybrid phenotypes that co-express epithelial and mesenchymal markers^2^, the concept of EMT/MET now includes partial transitions with intermediary phases^3^. Intermediate epithelial/mesenchymal phenotypes are found not only in vitro but also in vivo in different cancers, associated with greater tumor aggressiveness^4–7^.

The fluidity of EMT/MET suggests that regulatory circuits among transcription factors (TFs) involve complex interplays between EMT inducers (SNAI1/2, ZEB1/2, TWIST1, etc.)^8,9^ and EMT suppressors (GRHL2, OVOL1/2, etc.)^10,11^. Some TFs are key players in epigenetic remodeling such as DNA methylation and histone modification^12^. SNAI1, for example, recruits polycomb repressive complex 2 (PRC2) to the promoter of E-cadherin gene *CDH1*, which results in trimethylation of histone H3 lysine 27 (H3K27me3) and *CDH1* repression^13^. With the advancement of technologies, attempts have been made to elucidate genome-wide epigenetic changes during EMT, mainly using the TGF-β-induced^14-17^ or the TWIST1-induced system^18^. However, these studies lack the population to capture epigenetic changes associated with intermediate EMT states that occur during cancer progression, which may involve pathways independent of TGF-β or TWIST1. These different stages of cell-state transition may have distinct epigenetic regulations of epithelial/mesenchymal genes.

Ovarian cancer cells metastasize by shedding from primary tumors as free-floating aggregates in the ascitic fluids^19^ and this process involves EMT that allows cancer cells to overcome anoikis^20,21^. Our group demonstrated that ovarian cancer with an intermediate mesenchymal phenotype are more resistant to anoikis^7,22^. However, the regulation of EMT plasticity in ovarian cancer cells has remained elusive. Here, we study the epigenetic landscape of EMT involving intermediate states, using a previously established EMT scoring system^23^ and a panel of ovarian cancer cell lines with varying epithelial/mesenchymal phenotypes^7^. Our results show that epithelial genes are more subject to epigenetic reprogramming by CpG methylation and histone H3 modifications. These epithelial genes include *GRHL2* and the binding target genes of the encoded TF. We further demonstrate that EMT induced by GRHL2 knockdown would result in genome-wide epigenetic remodeling similar to that observed in ovarian cancer cells with progressive EMT phenotypes. GRHL2 overexpression and co-treatment of epigenetic-modifying drugs 5-azacitidine—an inhibitor of DNA methyltransferases (DNMTs), GSK126—an inhibitor of enhancer of zeste homolog 2 (EZH2), and/or mocetinostat—an inhibitor of class I histone deacetylases (HDACs), could induce MET to different extents, in cells lines with an intermediate EMT or a full EMT state.

## Results

### Differentially methylated CpGs occur at epithelial genes

**Fig. 1:**
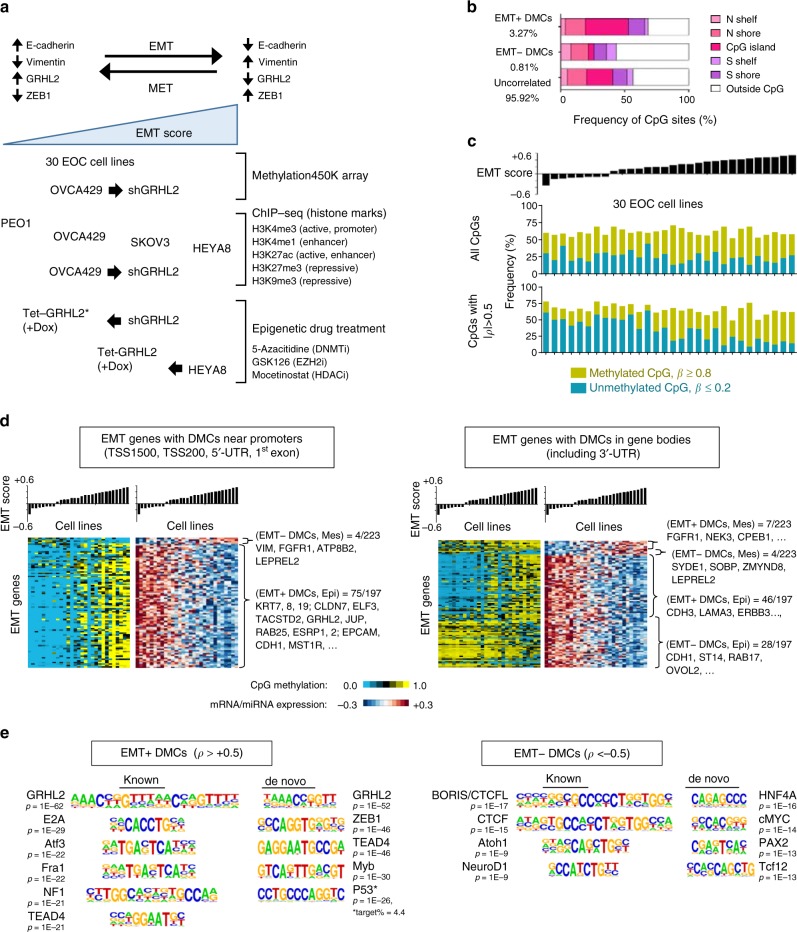
Identifying differentially methylated CpG sites (DMCs) across ovarian cancer cell lines with progressive epithelial–mesenchymal transition (EMT) phenotypes. **a** Diagram illustrates the EMT/mesenchymal–epithelial transition (MET) models used: 30 ovarian cancer cell lines with progressive EMT scores and OVCA429 GRHL2-knockdown model for Methylation 450K array; four-cell-line model (PEO1, OVCA429, SKOV3, HEYA8) and OVCA429 GRHL2-knockdown model for histone chromatin immunoprecipitation (ChIP)-sequencing; OVCA429 shGRHL2 Tet-GRHL2* (rescue) and HEYA8 Tet-GRHL2 for epigenetic drug treatment assays. **b** Bar charts indicate the percentage of EMT+ DMCs, EMT− DMCs, and non EMT-correlated CpG sites identified and their respective distribution in genome-wide CpG islands. EMT+ refers to a positive correlation with EMT; EMT− refers to a negative correlation with EMT. **c** Bar charts showing EMT score (top); frequency of methylated and unmethylated EMT-correlated DMCs (middle); and frequency of methylated and unmethylated EMT-correlated DMCs |*ρ*| > 0.5 (bottom) in 30 tested cell lines (*x*-axis). **d** Heatmaps of EMT signature genes with DMCs in promoter regions (left) and those with DMCs in gene bodies (right) showing CpG methylation level (blue = low; yellow = high) and the corresponding gene expression (red = high; blue = low) in ovarian cancer cell lines with progressive EMT scores (bar chart). Only genes with strong differential expression in correlation with EMT (|*ρ*| > 0.5) were shown. **e** Known motif enrichment analysis and de novo motif discovery showing DNA-binding motifs of transcription factors that are enriched at the DMCs, such as that of GRHL2 in EMT+ DMCs (*ρ* > 0.5, left panel) and CTCFL in EMT− DMCs (*ρ* <−0.5, right panel). EOC, epithelial ovarian carcinoma

From previous gene expression profiling of ovarian cancer cell lines, 306 mesenchymal and 213 epithelial signature genes were identified (Methods), the expression of which were used to generate an EMT score for each cell line (a higher EMT score indicates a more mesenchymal phenotype; a lower EMT score indicates a more epithelial phenotype)^23^. To identify CpG sites involved in EMT, we analyzed genome-wide CpG methylation of 30 ovarian cancer cell lines with progressive EMT phenotypes (Fig. 1a) using Infinium Human Methylation 450K array from Illumina. By correlating the methylation of each CpG with the EMT score of the cell line, we found that 5744 CpG sites (3.27%) were positively correlated with EMT (EMT+), while 1425 CpG sites (0.81%) showed a negative correlation (EMT−) (Fig. 1b, Supplementary Data 1). Among these EMT-correlated differentially methylated CpG sites (hereafter DMCs), 692 were associated with EMT signature genes. A higher percentage of EMT+ DMCs were located within CpG island (33.7%), as compared to the EMT− DMCs (4.5%) (Fig. 1b). These DMCs were distributed in promoter regions (including transcription start sites (TSS), 5′-untranslated region (UTR) and 1st exon), gene bodies, and intergenic regions (34–38%), with EMT+ DMCs occurring more frequently in promoter regions as compared to EMT− DMCs (35.8 vs. 26.4%) (Supplementary Fig. 1a). By designating CpG sites with *β* ≥ 0.8 as methylated and CpG sites with *β* ≤ 0.2 as unmethylated, we observed an overall methylation gain across the EMT gradient, especially in DMCs with strong EMT correlation (|*ρ*| > 0.5) (Fig. 1c). We validated the 450K methylation data with bisulfite pyrosequencing at selected loci of *CDH1* (E-cadherin gene) and *KRT19*, and the results showed an overall similar trend—the tested promoter regions were hypermethylated in cell lines with high EMT score (Supplementary Fig. 2).

Next, we cross-analyzed the methylation data with gene expression^23^. Among DMCs with strong EMT correlation (|*ρ*| > 0.5), 854 EMT+ DMCs and 218 EMT− DMCs were associated with differential gene expression (|*ρ*| > 0.5). Majority of the EMT+ DMCs (816/854, 95.6%) were in epithelial genes (gene expression correlated negatively with EMT), with the largest proportion found in promoter regions, followed by introns (Supplementary Fig. 1b, c, Supplementary Data 2). This was concordant with the conventional association between promoter methylation and gene silencing. Interestingly, most of the EMT− DMCs (194/218, 89.0%), although lesser in number, were also found in epithelial genes, but were predominantly inside gene bodies, especially introns (Supplementary Fig. 1b, c). Among EMT signature genes, a large proportion of epithelial genes (82/197, 41.62%), including *KRT7/8/19*, *GRHL2*, *ESRP1/2*, *EPCAM*, and *CDH1*, harbored EMT+ DMCs in promoters; 50 epithelial genes (25.38%), such as *CDH3*, *LAMA3*, and *ERBB3*, harbored EMT+ DMCs in gene bodies, while 31 epithelial genes (15.74%), including *CDH1*, *ST14*, and *OVOL2*, contained EMT− DMCs in gene bodies (Fig. 1d). Among 82 epithelial genes with EMT+ DMCs in promoters, 32 genes had additional EMT+ DMCs and 20 genes had EMT− DMCs in their gene bodies respectively (Supplementary Data 3). Hence, the role of methylated CpG in gene bodies could be different from their counterparts located in gene promoters, with respect to gene expression. In contrast to epithelial genes, very few mesenchymal genes contained DMCs. Only four mesenchymal genes (including *VIM*) harbored EMT− DMCs in promoters; seven contained EMT+ DMCs in gene bodies and four had EMT− DMCs in gene bodies (Fig. 1d, Supplementary Data 3). Therefore, in ovarian cancer, the expression of epithelial genes, but not mesenchymal genes, could be more susceptible to regulation by DNA methylation. Additional analyses showed that our results could be reproduced using gene sets defined by other studies, and the findings were applicable not only to ovarian cancer cell lines but also to clinical samples of ovarian and other cancer types (Supplementary Notes, Supplementary Fig. 3, Supplementary Data 4).

### The gain of CpG methylation after GRHL2 knockdown

TFs, which bind DNA with their unique recognition motifs, may recruit DNMTs to methylate CpG; or, they may have a protective role in preventing CpG methylation at their binding sites^24^. To identify TFs associated with the EMT-correlated DMCs, we performed motif analyses (known motif enrichment and de novo motif discovery). DNA-binding motifs of GRHL2, ZEB1, and E2A were found to be associated with the EMT+ DMCs, whereas DNA-binding motifs of CTCFL, CTCF, and HNF4A were associated with the EMT− DMCs (Fig. 1e). We utilized chromatin immunoprecipitation-sequencing (ChIP-seq) database ReMap2018 v1.2^25^ to perform enrichment analysis and the results also showed enrichment of EMT+ DMCs (including those at the TSS of genes) at DNA-binding sites of ZEB1 and GRHL2, whereas CTCFL and CTCF binding sites contained both EMT+ and EMT− DMCs (Supplementary Data 5). EMT+ DMCs that co-occurred with GRHL2 binding sites encompassed promoters and gene bodies, as well as intergenic regions (Supplementary Fig. 4).

**Fig. 2:**
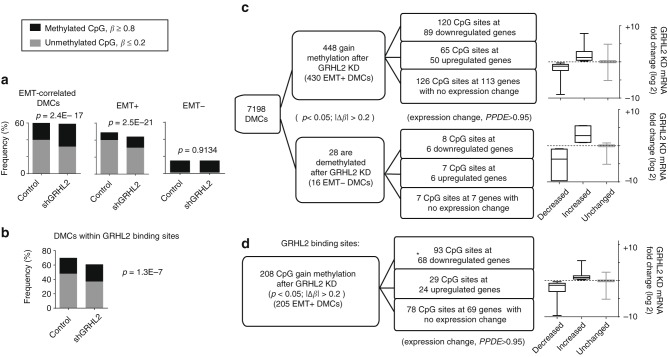
Gain in CpG methylation at epithelial–mesenchymal transition (EMT)-correlated differentially methylated CpG sites (DMCs) and GRHL2 binding sites following GRHL2 knockdown. **a** Bar graphs depict the frequency of methylated (*β* ≥ 0.8) and unmethylated (*β* ≤ 0.2) EMT-correlated DMCs (left), EMT+ DMCs (middle) and EMT− DMCs (right) in OVCA429 control vs. shGRHL2 cells. The *p* values of Fisher’s exact tests are shown for each group. **b** Bar graph indicates a slight increase (%) in methylated CpG (*β* ≥ 0.8) and a slight decrease (%) in unmethylated CpG (*β* ≤ 0.2) at DMCs within GRHL2 binding sites in GRHL2-knockdown cells, compared to control. The *p* value of Fisher’s exact test is shown. **c** Flow charts show the number of EMT-correlated DMCs that gain or lose methylation (*p* < 0.05; |∆*β*| > 0.2) after GRHL2 knockdown, with or without associated gene expression change. Box plots (right) depict the average expression log_2_ fold changes (messenger RNA (mRNA)) of the genes in each group after GRHL2 knockdown. **d** Flow chart shows the number of GRHL2 binding sites that gain or lose CpG methylation (*p* < 0.05; |∆*β*| > 0.2) after GRHL2 knockdown, with or without associated gene expression change. *This group is significantly enriched (93 vs. 29), based on Fisher’s exact test (*p* = 0.0427). Box-plot (right) depicts the average expression log_2_ fold changes (mRNA) of the genes in each group after GRHL2 knockdown. Error bars are ±s.e.m

As GRHL2 binds to promoters and enhancers of different epithelial genes^10,26^, our motif analyses suggested that GRHL2 may govern CpG methylation of epithelial genes at its binding sites. To address this, we performed 450K BeadChip array on control and GRHL2-knockdown OVCA429 cells that we previously generated. GRHL2 knockdown resulted in an intermediate state transition, with downregulation of E-cadherin and upregulation of vimentin^10^. Out of the 7198 EMT-correlated DMCs identified, 1673 sites changed in methylation after GRHL2 knockdown (*p* < 0.05), among which 448 gained methylation (∆*β* > 0.2), while only 28 were demethylated (∆*β* < −0.2) (Supplementary Data 6). An overall gain in methylated CpG sites (*β* ≥ 0.8) was seen only at the EMT+ DMCs (Fig. 2a). This methylation gain was also observed in GRHL2 binding sites after GRHL2 knockdown (Fig. 2b). CpGs that gained methylation were mostly found at 3′-UTR and gene bodies, and at the N shelves and S shores of CpG islands (Supplementary Fig. 5). Next, we integrated the CpG methylation data with gene expression profiling (RNA-sequencing (RNA-seq)). Out of 448 CpG sites that gained methylation in GRHL2-knockdown cells, 120 were associated with gene downregulations; 65 with gene upregulations; and 126 with no differential gene expression (Fig. 2c). In contrast, only seven CpG sites with six upregulated genes were demethylated in GRHL2-knockdown cells, including the mesenchymal gene *VIM* (encodes vimentin), which showed reduced CpG methylation in its promoter (Fig. 2c, Supplementary Data 6). Among CpG sites that gained methylation, almost half (208 sites) were found within GRHL2 binding sites, and majority of them (93 sites) were in downregulated genes (Fig. 2d), including *CLDN4*, *CGN*, *PROM2*, *S100A14*, *PVRL4* (methylated CpG at promoters), and *SPINT1* (methylated CpG at 3′-UTR) (Supplementary Data 6). These genes were considered epithelial specific as their expressions were negatively correlated with the EMT score (Supplementary Data 2). It has been reported that the functions of *CLDN4* and *CGN* are associated with tight junctions^27,28^, while *PVRL4* promotes cell–cell adhesion in ovarian cancer cells^29^. Although E-cadherin was downregulated after GRHL2 knockdown, no significant change in CpG methylation was observed. Overall, a subset of epithelial genes and GRHL2 targets showed differential CpG methylation (mainly methylation gain) after GRHL2 knockdown. As studies have suggested that the maintenance of unmethylated CpGs could involve cooperative binding of multiple activating TFs^24,30^, we speculate that GRHL2 could be one of them, associated with CpG sites of epithelial genes. Knockdown of GRHL2 alone would induce CpG methylation only in a subset of target genes, probably due to the presence of other methylation-inhibiting factors.

### MET effects of GRHL2 overexpression and DNMT inhibitor

**Fig. 3:**
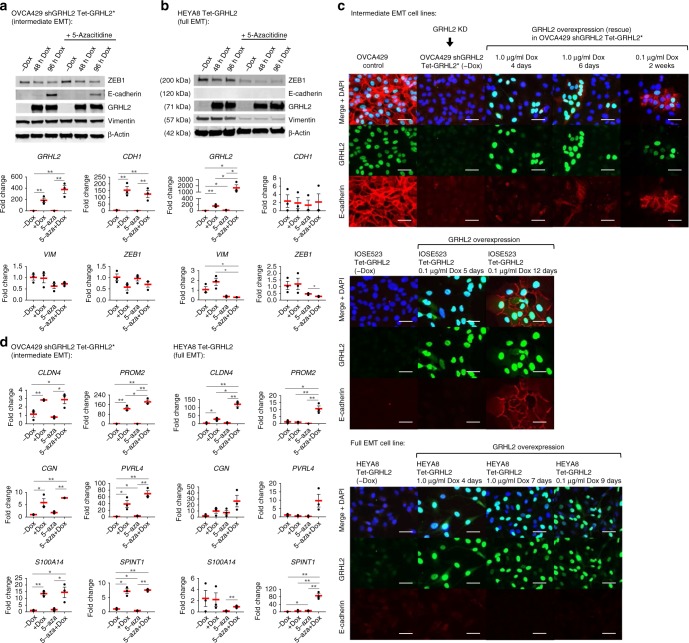
Effects of GRHL2 overexpression in combination with DNA methyltransferase (DNMT) inhibitor (5-azacitidine (5-aza)) treatment. **a** Western blots of ZEB1, E-cadherin, GRHL2, vimentin, and β-actin in OVCA429 shGRHL2 Tet-GRHL2* cells with/without doxycycline (Dox)-induced GRHL2 overexpression for 48 and 96 h, along with/without 5-azacitidine treatment. Representative blots from three independent experiments are shown. Bar graphs (bottom) showing messenger RNA (mRNA) fold changes of *GRHL2*, *CDH1*, *VIM*, *ZEB1* in OVCA429 shGRHL2 Tet-GRHL2* cells with/without 96 h doxycycline-induced GRHL2 overexpression, along with/without 5-aza treatment. Error bars = s.e.m. Unpaired *t* tests were used on independent triplicates, *0.01 < *p* < 0.05; **p < 0.01. **b** Same as in **a** but in the HEYA8 Tet-GRHL2 model. **c** Images of immunofluorescence staining showing GRHL2 (green) and E-cadherin (red) after GRHL2 knockdown or GRHL2 overexpression in the intermediate epithelial–mesenchymal transition (EMT) cell lines OVCA429 and IOSE523 and in the full EMT cell line HEYA8. Nuclei were stained blue (DAPI (4′,6-diamidino-2-phenylindole)). Scale = 50 μm. **d** Dot plots showing the mean mRNA fold changes (red lines) of *CLDN4*, *PROM2*, *CGN*, *PVRL4*, *S100A14*, *SPINT1* in OVCA429 shGRHL2 Tet-GRHL2* (left) and HEYA8 Tet-GRHL2 (right) with/without 96 h doxycycline-induced GRHL2 overexpression, along with/without 5-aza treatment (data from independent triplicates). Error bars = s.e.m. Unpaired *t* tests were performed: *0.01 < *p* < 0.05; **p < 0.01. KD, knock down

For GRHL2 overexpression, tetracycline-controlled transcriptional activation (Tet-On) system was used in OVCA429 shGRHL2 cells—to express mutant GRHL2* resistant to shGRHL2 (rescue) and in HEYA8 cells—to express wild-type GRHL2, upon doxycycline treatment. OVCA429 shGRHL2 cells with low E-cadherin and high vimentin had an intermediate EMT phenotype due to high levels of cytokeratins and an intermediate EMT score, whereas HEYA8 cells showed a full EMT phenotype with no E-cadherin, low cytokeratins, high vimentin, and high EMT score^7,10^. Interestingly, GRHL2 overexpression restored E-cadherin (more prominent at 96 h) in OVCA429 shGRHL2 cells, but not in the more mesenchymal HEYA8 cells (Fig. 3a, b). At time points beyond 6 days of GRHL2 induction, E-cadherin proteins were observed at cell–cell junctions, indicative of adherens junction formation (Fig. 3c). This time-dependent upregulation and localization of E-cadherin induced by GRHL2 were also observed in IOSE523, an immortalized ovarian surface epithelium cell line with low E-cadherin and an intermediate EMT phenotype, but not in HEYA8 (Supplementary Fig. 6, Fig. 3c). This suggested that ovarian cancer cells with a full EMT state could have different regulatory mechanisms compared to ovarian cancer cells with an intermediate EMT state. Since E-cadherin gene *CDH1* contained EMT+ DMCs that were methylated in HEYA8 and that some of the GRHL2 target genes, albeit small in proportion, could be subject to DNA methylation after GRHL2 knockdown, we tested the effects of 5-azacitidine, a DNA methylation inhibitor previously reported to upregulate E-cadherin^31,32^. In our models, 5-azacitidine treatment alone did not induce E-cadherin expression in either OVCA429 shGRHL2 or HEYA8 cells (Fig. 3a, b). The inefficacy of 5-azacitidine in HEYA8 could be due to partial demethylation (Supplementary Fig. 7). In combination with GRHL2 overexpression, 5-azacitidine also did not induce E-cadherin expression, but it further increased the *GRHL2* transcript levels (Fig. 3a, b). In addition, 5-azacitidine resulted in a reduction of vimentin and ZEB1, especially when combined with GRHL2 overexpression, in HEYA8 cells (Fig. 3b). Based on our GRHL2 ChIP-seq in ovarian cancer cells^10^, GRHL2 binds to its own promoter, the intron 2 enhancer of *CDH1*, intron 1 of *ZEB1*, but not to *VIM*. It is possible that the demethylation effects of 5-azacitidine contributed to the self-activation of GRHL2, which might regulate itself directly or indirectly via downregulation of its repressor ZEB1^33^.

We further examined the expression of genes that gained methylated CpGs and that were downregulated after GRHL2 knockdown—*CLDN4*, *PROM2*, *CGN*, *PVRL4*, *S100A14*, and *SPINT1*. Among these genes, *CLDN4*, *PROM2*, *CGN*, and *PVRL4* harbored GRHL2 binding sites at their promoter/5′-UTR regions^10^, which overlapped with their respective CpG sites that gained methylation, whereas *SPINT1* contained GRHL2 binding site at its 3′-UTR^10^ that overlapped with a CpG site that gained methylation after GRHL2 knockdown (Supplementary Data 6). Doxycycline-induced GRHL2 overexpression was able to restore the transcription of these genes in OVCA429 shGRHL2, but not in the more mesenchymal HEYA8 cells (except for *CLDN4*) (Fig. 3d). 5-Azacitidine treatment alone had no significant effects, but in combination with doxycycline-induced GRHL2 overexpression, 5-azacitidine significantly upregulated *CLDN4*, *PROM2*, and *SPINT1* in HEYA8 cells (Fig. 3d). Our data suggest that inhibiting DNA methylation might enhance the function of GRHL2 in activating the expression of certain target genes in ovarian cancer cells with a full EMT state.

### Remodeling of histone H3 at EMT genes across an EMT spectrum

**Fig. 4:**
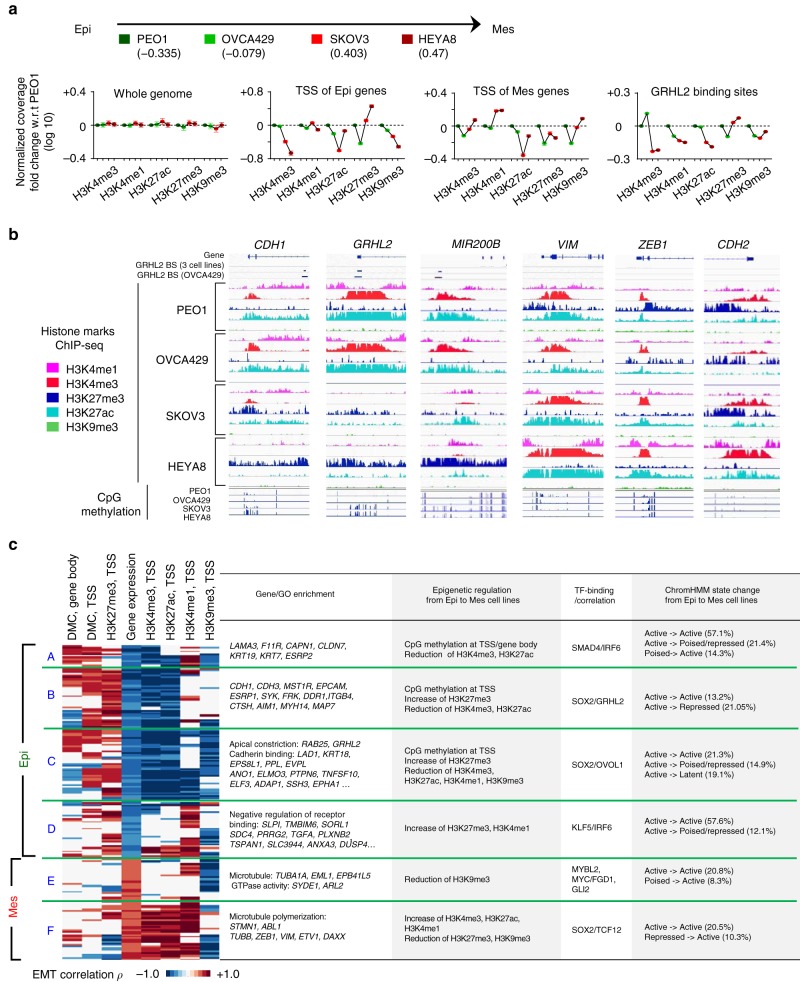
Differential histone modifications of epithelial–mesenchymal transition (EMT) genes across ovarian cancer cell lines with progressive EMT phenotypes. **a** Chromatin immunoprecipitation-sequencing (ChIP-seq) results (normalized log_10_ coverage; *y*-axis) of H3K4me3, H3K4me1, H3K27ac, H3K27me3, and H3K9me3 at the whole-genome level, transcription start sites (TSS) of epithelial (Epi) signature genes, TSS of mesenchymal (Mes) signature genes, and GRHL2 binding sites in PEO1, OVCA429, SKOV3, and HEYA8 cell lines, shown with their respective EMT scores. **b** The epigenetic landscape of Epi signature genes *CDH1*, *GRHL2*, *MIR200B* and Mes signature genes *VIM*, *ZEB1*, *CDH2* in PEO1, OVCA429, SKOV3, and HEYA8 cell lines. BS refers to binding site. **c** Heatmap depicts the EMT correlation (Pearson’s correlation *ρ*) of differentially methylated CpG sites (DMCs), histone H3 marks, and gene expression among 195 EMT signature genes that are clustered into six groups (A–F), in the four-cell-line model. Table (right) indicates gene names (with Gene Ontology analysis), combinations of epigenetic regulation, enrichment/correlation with TF binding, and ChromHMM state changes in each group of the genes

HEYA8 cells with a full EMT state responded to GRHL2 overexpression only upon DNA methylation inhibition. This suggested that epigenetic and chromatin accessibility is crucial for the EMT/MET plasticity. Besides DNA methylation, we study the histone modifications involved in EMT, by performing ChIP-seq of five important histone H3 modifications—H3K4me3 (marks active promoters), H3K4me1 (primed enhancers), H3K27ac (active enhancers), H3K27me3 (repressed transcription), and H3K9me3 (heterochromatin) on a four-cell-line model with progressive EMT scores: PEO1 (−0.335), OVCA429 (−0.079), SKOV3 (0.403), and HEYA8 (0.47), which showed decreasing expression of E-cadherin and increasing levels of vimentin. As expected, we observed higher levels of permissive marks (H3K4me3 and H3K27ac) and lower levels of repressive mark H3K27me3 at the TSS of epithelial signature genes, such as *CDH1*, *GRHL2*, and *MIR200B* cluster in cell lines with lower EMT score—PEO1 and OVCA429, as compared to cell lines with higher EMT score—SKOV3 and HEYA8 (Fig. 4a, b). The overall differences at the TSS of mesenchymal signature genes were less consistent, but still showed higher levels of H3K4me3 and H3K4me1, lower levels of H3K27me3 at mesenchymal genes *VIM*, *ZEB1*, and *CDH2* in the more mesenchymal SKOV3 and/or HEYA8 (Fig. 4a, b). At GRHL2 binding sites^10^, lower levels of active marks H3K4me3 and H3K27ac, while slightly higher levels of repressive mark H3K27me3 were observed in GRHL2-low/null cells (SKOV3 and HEYA8) vs. GRHL2-high cells (PEO1 and OVCA429) (Fig. 4a), which could be due to the majority of GRHL2 target genes being epithelial genes. Overall, the distribution of H3K4me3 was enriched near TSS and CpG islands, whereas the distributions of H3K4me1, H3K27ac, and H3K27me3 were more widespread covering not only promoters but also introns and intergenic regions (Fig. 4b, Supplementary Fig. 8a).

We performed ChromHMM analysis to interpret the combinatorial histone H3 modifications. Nine ChromHMM states were generated—1: primed enhancer (moderate H3K4me1 and low H3K27ac), 2: active enhancer (high H3K4me1 and H3K27ac), 3: active promoter-1 (high H3K4me3, H3K27ac and H3K4me1), 4: active promoter-2 (high H3K4me3 and H3K27ac), 5: poised/bivalent promoter (combination of H3K27me3, H3K4me3, H3K27ac, and H3K4me1), 6: PRC2-dependent repressed-1 (high H3K27me3), 7: PRC2-dependent repressed-2 (moderate H3K27me3), 8: latent/inactive (low or undetectable levels of all five tested histone marks), and 9: heterochromatin (moderate H3K9me3) (Supplementary Fig. 8b). States 1 to 4 were considered as active chromatin, while states 6, 7, and 9 were grouped as repressed chromatin. Alongside, unsupervised hierarchical clustering of EMT signature genes was performed based on differential DNA methylation (at TSS and gene body) and histone H3 modifications (at TSS), which revealed four clusters of epithelial genes (A–D) and two clusters of mesenchymal genes (E and F) with different combinations of epigenetic and potential TF regulation in correlation with EMT (Fig. 4c, Supplementary Data 7). Most of the genes showed a correlation of H3K4me3 with EMT, which indicates differential levels of promoter activity, but not all of them have significantly different ChromHMM states (Fig. 4c). EMT genes with differential CpG methylation and ChromHMM state include epithelial genes *ESRP1*, *ST14*, and *MAP7* (active to poised/repressed) from cluster B; and mesenchymal genes *FGFR1* and *VIM* (poised/repressed to active) from cluster F (Fig. 4c, Supplementary Data 7). Genes in cluster D and E showed little differences in H3K4me3 but with differential levels of H3K4me1 and H3K9me3, suggesting a possible remodeling of enhancers and heterochromatin (Fig. 4c). Genes in cluster A, C, and F showed differential levels in all five histone marks, suggesting that the regulation of these genes may involve remodeling of both promoter activity and enhancer/heterochromatic architecture during EMT (Fig. 4c). These results revealed various combinations of epigenetic regulations involving different TFs in the transcription of EMT genes.

### Remodeling of histone H3 after GRHL2 knockdown

**Fig. 5:**
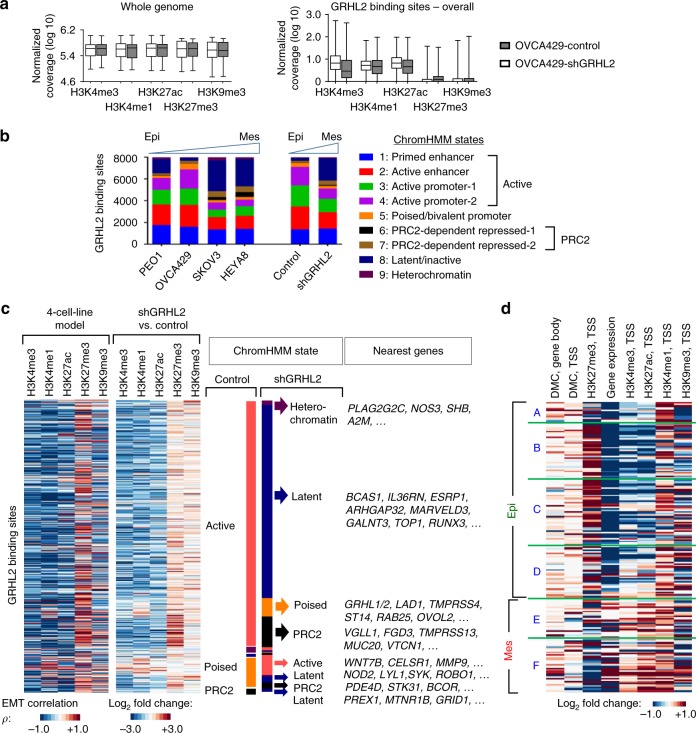
Histone modifications and chromatin states at GRHL2 binding sites and epithelial–mesenchymal transition (EMT) genes following GRHL2 knockdown. **a** Box plots showing chromatin immunoprecipitation-sequencing (ChIP-seq) results (normalized log_10_ coverage) of H3K4me3, H3K4me1, H3K27ac, H3K27me3, and H3K9me3 at the whole-genome level (left) and GRHL2 binding sites (right) in OVCA429 control and shGRHL2 cells. The band within the box represents the median and the whiskers indicate the minimum to the maximum. **b** Bar chart shows the number of GRHL2 binding sites with nine different ChromHMM states in the four-cell-line EMT model (PEO1, OVCA429, SKOV3, HEYA8) and GRHL2-knockdown EMT model (OVCA429 control vs. shGRHL2). **c** Heatmaps showing GRHL2 binding sites with differential histone H3 modifications in correlation with EMT represented by the four-cell-line model (left, Pearson’s correlation *ρ*), and in the GRHL2-knockdown model (right, log_2_ fold change). GRHL2 binding sites have lower levels of H3K4me3, H3K4me1, H3K27ac (blue) and higher levels of H3K27me3 (red) in EMT score-high and shGRHL2 cells, compared to EMT score-low and OVCA429 control cells, respectively. Diagram next to heatmaps indicates ChromHMM states and the nearest genes of the respective GRHL2 binding sites in OVCA429 control and shGRHL2 cells. **d** Heatmap depicts differential levels (log_2_ fold change) of methylation at differentially methylated CpG sites (DMCs), histone H3 marks, and gene expression among 195 EMT signature genes (shown in Fig. 4c) in OVCA429 shGRHL2 vs. control cells

ChIP-seq of the five histone H3 modifications was carried out on control and GRHL2-knockdown OVCA429 cells. At GRHL2 binding sites, GRHL2 knockdown resulted in an overall decrease in active marks (H3K4me3 and H3K27ac) and a slight gain in repressive mark H3K27me3 (Fig. 5a). Based on ChromHMM analysis (Supplementary Fig. 8b), the GRHL2 binding sites in shGRHL2 cells showed a significant decrease in active chromatin states (active enhancers and active promoters), accompanied by an increase in latent, poised, and repressed chromatin states, which resembled the states observed in SKOV3 and HEYA8 from the four-cell-line model (Fig. 5b). Most of these state changes were from an active to a latent/inactive state (21%, 1703) (Supplementary Fig. 9), found mainly at intronic or intergenic regions, and they encompassed genes such as *BCAS1*, *IL36RN*, *ESRP1*, and *MARVELD3*, which were downregulated (Fig. 5c, Supplementary Data 8). GRHL2 binding sites that changed from an active state to a poised/bivalent state include *GRHL1*, *GRHL2*, *LAD1*, *ST14*, *RAB25*, *OVOL2*, whereas GRHL2 binding sites that switched from an active state to a PRC2-repressed state include *VGLL1*, *FGD3*, *TMPRSS13*, and *MUC20* (Fig. 5c, Supplementary Data 8). Strikingly, these state transitions (mostly active to latent) were similar to that observed in the four-cell-line model (Supplementary Fig. 9). Besides GRHL2 binding sites, some of the epithelial signature genes that were downregulated after GRHL2 knockdown also showed histone changes at their respective TSS (Fig. 5d), resembling changes observed in the four-cell-line model (Fig. 4c). Using the OVCA429 shGRHL2 Tet-GRHL2* model, we performed ChIP-quantitative PCR (qPCR) on a few GRHL2 binding sites and showed that GRHL2 re-expression led to reduced H3K27me3 at gene promoters (*GRHL2* and *OVOL2*) and increased H3K4me1 and H3K27ac at proximal/distal enhancers (intron 8 of *GRHL2* and intron 2 of *CDH1*) (Supplementary Fig. 10a–c). This suggested that GRHL2, as an epithelial-specific TF, might act to repel gene repressors, such as the PRC2 complex and the HDACs, at promoters and enhancers of its target genes.

### EZH2 inhibitor and HDAC inhibitor enhance GRHL2-mediated MET

**Fig. 6:**
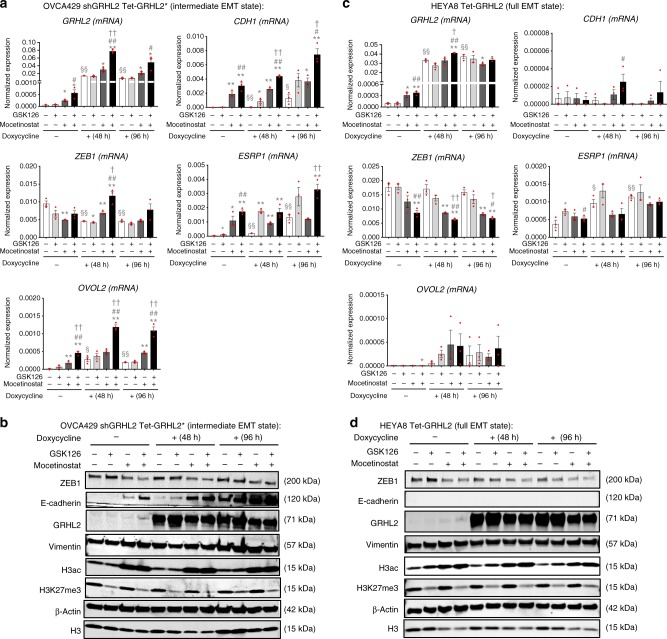
Effects of GSK126 (enhancer of zeste homolog 2 (EZH2) inhibitor) and mocetinostat (histone deacetylase (HDAC) inhibitor) in combination with GRHL2 overexpression. **a** Normalized messenger RNA (mRNA) expression (2^−ΔCt^) of *GRHL2*, *CDH1*, *ZEB1*, *ESRP1*, and *OVOL2* in OVCA429 shGRHL2 Tet-GRHL2* cells with/without doxycycline treatment (to induce GRHL2 expression) for 48 or 96 h, with/without GSK126 (EZH2 inhibitor) and/or mocetinostat (HDAC inhibitor). Data of independent triplicates are shown (red dots). Unpaired *t* tests were performed: * represents significance of histone drug-treated vs. no histone drug treatment; ^#^ represents significance of two drugs combined vs. GSK126 treatment only; ^†^ represents significance of two drugs combined vs. mocetinostat treatment only; ^§^ represents significance of doxycycline-treated vs. no treatment control (one symbol: 0.01 < *p* < 0.05; two symbols: *p* < 0.01). Error bars = s.e.m. **b** Western blots of ZEB1, E-cadherin, GRHL2, vimentin, H3ac, H3K27me3, β-actin, and total H3 in OVCA429 shGRHL2 Tet-GRHL2* cells with/without doxycycline treatment (to induce GRHL2 expression) for 48 or 96 h, with/without treatment of GSK126 and/or mocetinostat. Representative blots from three independent experiments are shown. **c** Same as in **a** but in HEYA8 Tet-GRHL2 cells. **d** Same as in **b** but in HEYA8 Tet-GRHL2 cells

As GRHL2 knockdown resulted in increased H3K27me3 and reduced H3K27ac at epithelial genes, we went on to test the MET effects of EZH2 inhibitor GSK126—which could reduce the global levels of H3K27me3, and HDAC inhibitor mocetinostat—which could increase the global levels of acetylated histone H3 (H3ac) (Fig. 6b, d), in the GRHL2 overexpression models. In OVCA429 shGRHL2 Tet-GRHL2* cells with an intermediate EMT phenotype, doxycycline-induced GRHL2 expression resulted in time-dependent upregulation of not only *CDH1* but also epithelial genes *ESRP1* and *OVOL2* (Fig. 6a, b). GSK126 alone did not restore *CDH1*/E-cadherin expression, but it enhanced the upregulation of *CDH1*/E-cadherin and *ESRP1* induced by GRHL2, whereas mocetinostat alone was sufficient to upregulate significantly *CDH1*/E-cadherin and *ESRP1*, marginally *GRHL2* (messenger RNA (mRNA) and protein) and *OVOL2* (Fig. 6a, b). GSK126 and mocetinostat co-treatment showed stronger effects compared to single treatments, and when combined with GRHL2 overexpression, GSK126 and mocetinostat co-treatment resulted in even greater upregulations of these epithelial genes (Fig. 6a, b). There was no significant change in the expression of mesenchymal marker vimentin, but we observed ZEB1 downregulation mediated by mocetinostat treatment (Fig. 6a, b). We repeated the experiments in another cell line with an intermediate EMT phenotype—IOSE523 Tet-GRHL2 and the results were consistent with that observed in OVCA429 shGRHL2 Tet-GRHL2*, with a more significant ZEB1 downregulation (mRNA and protein) mediated by GRHL2 overexpression (Supplementary Fig. 11). Therefore, in ovarian cancer cells with intermediate phenotypes, GRHL2 overexpression resulted in partial MET from an intermediate mesenchymal towards an intermediate epithelial state, with the co-expression of E-cadherin and vimentin. These effects could be enhanced by EZH2 inhibition and HDAC inhibition.

In contrast, HEYA8 Tet-GRHL2 cells with a full EMT phenotype showed different results. At the transcript level, epithelial genes were barely affected—GSK126 alone upregulated *ESRP1*, whereas mocetinostat alone upregulated *GRHL2* (Fig. 6c). Overexpression of GRHL2 induced *ESRP1* expression, whereas the combination with GSK126 and/or mocetinostat did not show any additive effect (Fig. 6c). These transcripts with marginal increments only translated to very minimal protein expression (e.g., GRHL2 in Dox-off cells) (Fig. 6d). Interestingly, there was a consistent downregulation of ZEB1 (mRNA and protein), and the effect was strongest in the combination of GRHL2 overexpression, GSK126, and mocetinostat (Fig. 6c, d). Overall, MET effects of GRHL2 overexpression, EZH2 inhibition, and HDAC inhibition were limited in ovarian cancer cells with a full EMT state, as these cells may have additional mechanisms that would hinder the expression of epithelial genes.

**Fig. 7:**
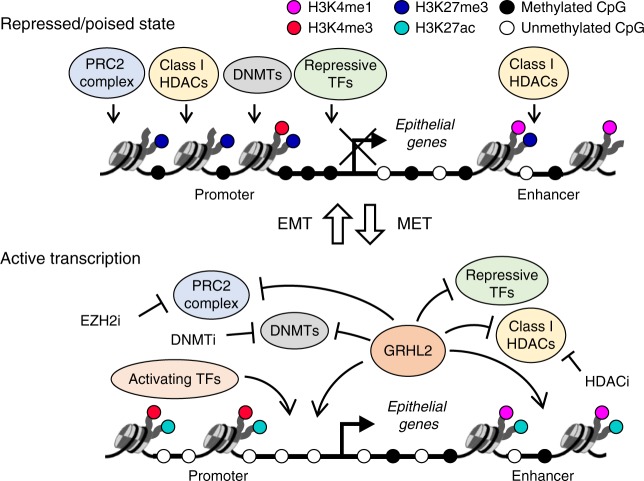
Interplay of GRHL2 and epigenetic modifiers in CpG methylation and nucleosomal remodeling of epithelial genes. A schematic model illustrating the epigenetic regulation of epithelial genes in the repressed/poised (top) or active (bottom) state during epithelial–mesenchymal transition/mesenchymal–epithelial transition (EMT/MET) involving intermediate phenotype changes. CpG methylation at promoters are associated with gene repression whereas CpG methylation in gene bodies may or may not be associated with gene transcription. A repressed/poised promoter has high H3K27me3 and low H3K4me3, whereas an active promoter has high H3K4me3 and high H3K27ac. A repressed/poised enhancer region is characterized by H3K4me1 with/without H3K27me3, whereas an active enhancer is characterized by H3K4me1 with high H3K27ac. GRHL2 may inhibit the activities of repressive TFs and/or epigenetic repressors, such as polycomb repressive complex 2 (PRC2) complex, histone deacetylases (HDACs) and DNA methyltransferases (DNMTs) at promoters and/or enhancers of epithelial genes. Reciprocally, epigenetic modifiers may modulate the function of GRHL2 in maintaining/activating the expression of epithelial genes

Collectively, our results suggest that different combinations of epigenetic remodeling (histone modifications, CpG methylation, or both) are involved in the repression/activation of epithelial genes during EMT/MET involving intermediate states, and GRHL2, by itself, may induce only a part of them (Fig. 7). Reciprocally, the activation of epithelial genes requires not only the modification of the epigenetic landscape but also the presence of activating TFs such as GRHL2, which may further form a positive feedback regulation to maintain the permissive chromatin around epithelial genes.

## Discussion

Cancer cells display EMT/MET plasticity in the form of intermediate/hybrid/metastable states with co-existing epithelial and mesenchymal features^2^. Our findings here suggest that, across the EMT spectrum demonstrated in ovarian cancer cell lines, epithelial genes are more subject to epigenetic regulation, with different clusters of genes showing reduction in active histone marks H3K4me3 and H3K27ac; increase in repressive histone mark H3K27me3; substantial gain in CpG methylation at TSS; and a mixed gain/loss of CpG methylation within gene bodies. The higher occurrence of DNA methylation gain along the EMT gradient, especially in gene promoters, is consistent with previous studies: TGF-β induced EMT results in hypermethylated DNA loci;^15^ a higher number of hypermethylated CpG sites are found in mesenchymal-like lung cancer cells than in the epithelial-like phenotype;^34^ DNA hypermethylation is more prominent at gene promoters during TGF-β-induced EMT in Madin–Darby canine kidney (MDCK) cells^16^. Besides promoter hypermethylation, concurrent DNA hypomethylation at the global level;^18^ or specifically at promoters^35^ and gene bodies^16^ resulted from EMT has also been documented—the latter coincides with our finding that CpG methylation within gene bodies (non-promoter) may correspond to either gene expression or gene repression. In our EMT models, only a few mesenchymal genes with upregulated expression show reduced CpG methylation at TSS (*VIM* and *FGFR1*). The lack of DMCs in mesenchymal genes suggests that DNA methylation is unlikely the main repressive mechanism of these genes in ovarian cancer cells with lower EMT scores. Without the epigenetic barrier of DNA methylation, ovarian cancer cells with epithelial features may have a higher level of plasticity in switching on mesenchymal genes during EMT, compared to cells with mesenchymal features in switching on epithelial genes during MET. Indeed, bivalent H3K4me3 and H3K27me3 marks are observed at the promoters of mesenchymal genes (indicative of a repressed but poised mode) in the more epithelial PEO1 and OVCA429 cells. Some of these bivalent marks are replaced by active states in the more mesenchymal SKOV3 and HEYA8 cells as the H3K27me3 mark is reduced.

Epigenetic remodeling during EMT/MET is likely to involve various epigenetic modifiers, while the site specificity of these modifiers may depend on DNA-binding factors such as EMT-TFs. Among these TFs, SNAI1 and ZEB1 are known to repress E-cadherin, through the recruitment of DNMTs^36,37^ and/or the PRC2 complex^13^. In our study, we show that CpG sites that gain methylation in correlation with EMT are enriched not only at the DNA-binding sites of EMT-inducing TF ZEB1 but also at the DNA-binding sites of EMT-suppressing TF GRHL2. GRHL2 knockdown results in CpG methylation gains and ChromHMM histone state changes from active to latent, poised/bivalent, or repressed in epithelial genes, including genes with GRHL2 binding sites. These changes recapitulate the epigenetic transitions from ovarian cancer cells with lower EMT score (GRHL2-high) to those with higher EMT score (GRHL2-low). Therefore, our data suggest GRHL2 as a potential epigenetic remodeler of epithelial genes that employs both DNA methylation and histone modifications. These results support recent findings, which posit GRHL2 and other Grainyhead family members as pioneer factors that regulate the chromatin accessibility of epithelial enhancers^38,39^, but this execution may depend on the existing state of the cell lines. Our previous and current studies also indicate the possibility of self-activation of GRHL2, through direct binding at its own promoter and/or indirect downregulation of its repressor ZEB1 via activation of miR-200. These findings support the role of GRHL2 as a “phenotypic stability factor” suggested by Jolly et al.^11^ (but with mir-200-activating and self-activating properties) that may stabilize the hybrid epithelial/mesenchymal phenotype associated with collective cell migration. As self-activation of TFs has been implicated in cell-fate commitment^40^, we propose an idea of GRHL2 being a pioneer factor that may activate itself and its homolog GRHL1 (GRHL2 ChIP-seq peaks are found in *GRHL1* promoter and gene body), and through binding at target regions, may increase the transcriptional accessibility of epithelial genes for the maintenance of a stable epithelial or an intermediate/hybrid epithelial phenotype.

In relation to histone modification, GRHL2 has been reported to impair keratinocyte differentiation through downregulation of epidermal differentiation genes, resulting in reduced binding of histone demethylase Jmjd3 and elevated H3K27me3 at their promoters^41^. In MDCK cells, GRHL2 directly binds to and inhibits histone acetyltransferase (HAT) p300, and hence silences mesenchymal genes required for tubulogenesis^42^. However, these proposed histone-modifying functions of GRHL2 in gene repression may not explain its role in epithelial gene activation observed in our study. Being part of the PRC2 complex, EZH2 is an important repressor of E-cadherin^13,43^. Conflicting reports show that inhibition of EZH2 leads to E-cadherin restoration in endometrial cells^44^, but not in ovarian cancer cells^45^. Consistent with the latter finding, we demonstrate that inhibiting EZH2 by GSK126 is not sufficient to restore epithelial gene expression in ovarian cancer, even though H3K27me3 is reduced. Note that the levels of H3K27me2 and H3K27me1 have not been checked in this study, and these markers may hinder epithelial gene transcription. However, in the presence of GRHL2, which binds to intronic enhancers of *CDH1* and *ESRP1*, GSK126 treatment could enhance, albeit limited, initial transcription of these genes. This suggests that GRHL2 might play a role in repelling the PRC2 complex from its target epithelial genes. On the other hand, treatment of mocetinostat alone is sufficient to induce expression of epithelial genes especially E-cadherin, suggesting that class I HDACs (mocetinostat inhibits the activity of HDAC1, 2, 3, and 11) could be key epithelial gene repressors that counteract GRHL2. In fact, the DNA-binding sites of HDAC2 overlap significantly with those of GRHL2 (ChIP-seq data, Supplementary Data 9). Moreover, unlike HDAC1, 3, and 6 that are associated mainly with promoters, HDAC2 binds to promoters and also intergenic enhancers^46^, a feature shared by GRHL2. Thus far, we did not detect any physical binding between GRHL2 and HDAC2. One potential co-factor of GRHL2 identified via co-immunoprecipitation-mass spectrometry is Ki67 (Supplementary Data 10), which has been reported to interact with epigenetic regulators HDAC2 and SUZ12 at heterochromatins^47^. If the GRHL2-Ki67 interaction is true, GRHL2 could possibly act as a “scaffold protein” of epithelial genes to insulate the activity of Ki67-associated gene repressors. Altogether, these findings posit a potential involvement of GRHL2 in the HAT/HDAC and PRC2 complex dynamics associated with epithelial gene regulation, which demands further validation.

GRHL2 overexpression in combination with epigenetic-modifying drugs GSK126 and mocetinostat can induce epithelial gene expression only in OVCA429 shGRHL2 and IOSE523 cells with intermediate phenotype, but not in the full mesenchymal HEYA8 cell. This is probably due to DNA hypermethylation at the epithelial gene promoters in cells with full EMT state. Reducing DNA methylation by DNMT inhibition could induce MET, as ZEB1 and vimentin were downregulated in HEYA8 upon 5-azacitidine treatment. Moreover, 5-azacitidine could enhance the function of GRHL2 in activating epithelial genes such as *CLDN4*, *PROM2*, and *SPINT1* in HEYA8 cells. These genes are among those that showed reduced expression and CpG methylation gain in ovarian cancer cells with high EMT score (including HEYA8) and also in GRHL2-knockdown cells. It remains unclear whether these CpG methylation gains are due to the functions of SNAI1 and ZEB1. The specific DNMTs or TET enzymes involved are also not known. DNMT3B could be an interesting candidate as the intragenic EMT+ DMCs in epithelial genes were enriched in DNMT3B binding, based on available ChIP-seq data (Supplementary Fig. 1d). Meanwhile, DNMT3A has been highlighted as a key repressor of epithelial genes, including *CDH1* and *GRHL2*, in a prostate cancer-associated fibroblast-induced EMT model^35^. Since GRHL2 has been reported to hinder the activity of DNMT1 at the CpG island of the telomerase gene (*TERT*)^48^, the possibility of GRHL2 counteracting DNMTs in the regulation of epithelial genes deserves future investigation. For key epithelial marker E-cadherin, ovarian cancer cells with a full EMT state may have tighter controls than ovarian cancer cells with an intermediate EMT state. Neither DNA methyltransferase inhibitor 5-azacitidine nor its combination with GRHL2 overexpression could induce E-cadherin in HEYA8 cells. Other combinations were attempted, but to no avail: 5-azacitidine with GSK126 showed similar negative results (Supplementary Fig. 12), whereas 5-azacitidine with mocetinostat resulted in extensive cell death. As the currently available epigenetic-modifying drugs such as 5-azacitidine, GSK126, and mocetinostat are limited by non-specificity or/and cellular toxicity^49^, novel targeted epigenome editing using the CRISPR/Cas9 system^50^ will be needed to improve functional validation of locus-specific epigenetic modifications. Transcription of epithelial genes such as E-cadherin gene *CDH1* may also depend on three-dimensional chromatin interactions, as GRHL2 binds to the intronic enhancer of E-cadherin that forms a chromatin loop with the E-cadherin promoter^51^. With the recent evidence of GRHL2 being required for cohesin binding^38^, we speculate that GRHL2, which binds to many other promoters and distal enhancers, could act as an anchor for long-range DNA interactions among other epithelial genes. These enhancer–promoter loopings and/or chromatin higher order folding around epithelial genes might be altered in GRHL2-null cells with full EMT state, as these cells have lower plasticity for MET to occur. In sarcomas, downregulation of ZEB1 or the ZEB1-associated chromatin remodeler BRG1 is required for GRHL2-mediated activation of E-cadherin expression^52^, suggesting that ZEB1-associated chromatin remodeling is an important hindrance to GRHL2. However, in our full EMT cell line HEYA8, GRHL2 overexpression coupled with ZEB1 downregulation through ZEB1 knockdown or miR-200 overexpression is not sufficient to upregulate E-cadherin (Supplementary Fig. 13). 5-Azacitidine treatment in conjunction with GRHL2 overexpression downregulates ZEB1 and induces *CLDN4*, *PROM2*, and *SPINT1* but not E-cadherin. Therefore, the tight repression of E-cadherin in full mesenchymal ovarian carcinoma cells such as HEYA8, which may involve ZEB1-independent nucleosome assembly or chromatin rearrangements that hinder GRHL2 binding, remains to be elucidated. DNase sensitivity, FAIRE, ATAC-seq, or chromatin capture assays coupled with GRHL2 ChIP experiments will be useful to examine the interplay of GRHL2 and chromatin accessibility of epithelial genes in ovarian carcinoma cells with an intermediate or a full EMT phenotype. As cell lines may comprise heterogeneous populations, analyses of EMT markers, EMT-driving/suppressing TFs, and epigenetic modifications at the single-cell level through fluorescence-activated cell sorting sorting will provide a more precise elucidation of EMT plasticity in future studies.

## Methods

### Cell culture

The 30 ovarian cancer cell lines used were cultured as previously described^7,10^. The EMT score and disease origin of each cell line (Cellosaurus database)^53^ are listed in Supplementary Table 1, along with indications of their usage in three reported studies^54-56^. Stable short hairpin RNA control and shGRHL2 (shGRHL2 #12) OVCA429 cells were from our previous study^10^.

### Derivation of EMT signature genes

In our previous study, we categorized a panel of ovarian cancer cell lines into epithelial or mesenchymal phenotype based on the difference between E-cadherin and N-cadherin positivity on the cell surface by immunostaining described^7^. From this, we generated a preliminary EMT signature using BinReg, and predicted the EMT phenotype on 142 ovarian cancer cell lines. The cell lines with the highest probabilities for epithelial or mesenchymal phenotype were selected and used to identify the EMT signature genes through SAM (*q* value = 0) and ROC (threshold >0.85). This EMT signature contained canonical EMT markers such as *CDH1*, *GRHL2*, *EPCAM*, *CDH2*, *ZEB1*, and *VIM*.

### Tet-On GRHL2 overexpression and epigenetic drug treatments

Lenti-X Tet-On 3G-Inducible Expression System (Clontech) was used for GRHL2 overexpression. The complementary DNA (cDNA) of GRHL2 was cloned into pLVX-TRE3G vector (631191, Clontech) using standard molecular cloning techniques. The mutated GRHL2* (resistant to shGRHL2 #12) was generated by introducing four silent point mutations to the wild-type cDNA using QuickChangeII XL Site-Directed Mutagenesis Kit (Agilent). The 293T cells were transfected with pLVX-Tet3G, viral packaging mix, and pLVX-TRE3G-GRHL2 or pLVX-TRE3G-GRHL2* using Lenti-X HTX Packaging Mix 2 System (631260, Clontech). Viruses were harvested to infect IOSE523, HeyA8, and OVCA429 shGRHL2 #12 cells. GRHL2 expression was induced by doxycycline (1 μg/ml for 48 or 96 h). Cells were treated with GSK126 (S7061, Selleck Chemicals) at a final concentration of 5 μM for 72 h; mocetinostat (S1122, Selleck Chemicals) at 1 μM (OVCA429 shGRHL2 Tet-GRHL2*) or 0.5 μM (IOSE523 and HeyA8) for 48 h; 5-azacitidine (S1782, Selleck Chemicals) at 1 μM for 144 h.

### DNA methylation analysis

Genomic DNA of 31 ovarian cancer cell lines, with duplicates, were purified using standard phenol–chloroform DNA extraction or AquaPure Genomic DNA Kit (732-6340) from Bio-Rad. The samples were profiled using Infinium HumanMethylation450K BeadChip array (Illumina, CA, USA). The DNA methylation data were processed and normalized using R version 3.1.2, Bioconductor 2.6 ChAMP 1.2.8. PBC normalization was applied. Probes not detected (*p* > 0.05) and probes reported as cross-reactive were excluded from analysis^57^. All samples passed quality checks by MethylAid 1.1.0. Inter-replicates methylation correlation and unsupervised hierarchical clustering showed strong correlation among replicates. One outlier OV90 (with low yield) was removed from analyses. No apparent batch effect due to potential confounding factors was observed on the normalized data. The normalized *β* values were averaged across replicates prior to downstream analysis. To increase statistical power, probes with low variation (standard deviation <0.05), interquartile range <0.1, or methylation level difference <0.1 in methylated and unmethylated control, were removed. The final dataset has 30 ovarian cancer cell lines and 175,515 CpG sites, annotated using HumanMethylation450_15017482_v1-2. Additional annotations were from Homer v4.7.2^58^, hg19. Chromatin states and functional domains were obtained from Epigenome Roadmap (http://egg2.wustl.edu/roadmap/web_portal/chr_state_learning.html#core_15state) and ENCODE (http://genome.ucsc.edu/ENCODE/downloads.html). EMT-correlated DMCs were identified using Spearman’s correlation coefficient test between DNA methylation levels (*β* values) and EMT scores^23^. Thresholds of *β* ≥ 0.8 for methylated CpG and *β* ≤ 0.2 for unmethylated CpG were applied based on the *β* distribution of the *β* value normalization and were verified to be suitable to differentiate Illumina’s methylated control from the unmethylated control (Supplementary Fig. 14). DNA methylation data were cross-analyzed with gene expression (Affymetrix Gene ST array) for all cell lines except HEYA8 (gene expression data from Affymetrix U133A). Correlations with *ρ* >+0.5 and <−0.5 were deemed significant.

### Histone ChIP-sequencing and ChIP-qPCR

Cells were cross-linked by 1% formaldehyde (10 min), sonicated, and used for chromatin immunoprecipitation as described^10^. Antibodies used include rabbit immunoglobulin G (sc-2027, Santa Cruz), anti-H3K4me1 (ab8895, Abcam), anti-H3K4me3 (CS-003-100, Diagenode), anti-H3K27me3 (07-449, Millipore), anti-H3K27ac (ab4729, Abcam), and anti-H3K9me3 (ab8898, Abcam). Purified samples and input controls were used for next-generation sequencing or qPCR. ChIP-seq libraries were constructed from the ChIP-DNA using Illumina TruSeq ChIP Sample Prep Kit (IP-202-1012), with 15 to 18 cycles of PCR amplification. DNA size selection was performed using AMPure XP beads. For ChIP-qPCR, the primers used are listed in Supplementary Table 2.

### ChIP-seq data processing and analysis

The 75-bp single-end sequencing was performed using Illumina NextSeq 500 platform. Quality of the dataset was assessed based on ENCODE ChIP-seq guidelines^59^ using FastQC v0.11.1. Raw reads were mapped to the UCSC hg19 reference human genome using Novoalign v3.02.06 with default parameter settings. Alignment rates were between 78.3% and 96.1%. Only uniquely mapped reads were retained for analysis. Post filtering, roughly 20–60 million unique reads were obtained for each histone modification in each condition. Strand cross-correlation analysis was performed according to ENCODE Irreproducible Discovery Rate pipeline using phantompeakqualtools v1.1. All samples had acceptable read quality, mapping statistics, library complexity, and strand cross-correlation analysis quality. Format conversions of the files were done using samtools v0.1.19 and bedtools v2.25.0. Mapped reads were pooled across replicates and peaks were identified by comparing the pooled ChIP-seq data against the pooled control using MACS2 v2.1.0.20150731 for each histone modification in each condition, with *p* = 0.001 (−p) and “–to-large” flag. Diffuse histone marks H3K27me3 and H3K9me3 were called with additional flag “--broad". Visualization was done using IGV v2.3.68.

### Combinatorial chromatin states prediction

ChromHMM v1.1 was used to derive combinatorial chromatin states in PEO1, OVCA429, SKOV3, HEYA8, OVCA429 control and OVCA429 shGRHL2 cells. All five histone H3 marks (H3K4me3, H3K4me1, H3K9me3, H3K27me3, and H3K27ac) were used to train the model. Reads from replicate data were pooled and binarized by comparing ChIP-seq read counts to whole-cell extract control using BinarizeBed. Using LearnModel function, several HMM models (with 5 to 20 states) were trained. A model with nine states was used.

### Unsupervised hierarchical clustering of epithelial and mesenchymal genes

EMT signature genes^23^ with a strong correlation with EMT score (Spearman’s |*ρ*| >0.5) and with coverage in both Infinium methylation 450K and histone marks were analyzed. For genes with multiple DMCs near TSS (probesets annotated as TSS1500, TSS200, 5′-UTR, and 1st exon) or in gene bodies (probesets annotated as body and 3′-UTR), only DMCs with the strongest EMT–methylation correlation were shown. For histone marks, annotation by Homer v4.7.2^58^ and hg19 was used. Pearson’s correlation *ρ* was subjected to hierarchical clustering using Cluster3.0 (http://bonsai.hgc.jp/~mdehoon/software/cluster/software.htm). Enrichr^60^ was used in the enrichment analysis of TF binding/correlation.

### Motif analysis

Motif enrichment and de novo motif discovery were computed using Homer v4.7.2^58^, on the genomic regions with EMT-correlated DMCs. Default parameters were used. Motifs discovered were subjected to filters of motif length ≥5, alignment must include part of the core motif, sufficient complexity, not simple repeat, and target sequenced described >5%.

### RNA-sequencing

RNAs of OVCA429 control and shGRHL2 cells were subjected to RNA paired-end sequencing. The quality of RNA-seq data was assessed using FastQC v0.11.5 (http://www.bioinformatics.babraham.ac.uk/projects/fastqc/) and RNASeQC v1.1.8^61^. Quality metrics were at an acceptable range. Sequences were mapped to human genome hg19 using STAR v2.4.2a^62^ and transcripts were quantified using RSEM 1.2.25^63^ with Gencode v19 annotation. All samples have an average read length of 150 and a unique mapping rate >90% (average 56 million unique reads). EBseq v1.20.0^64^ was used to identify differentially expressed genes.

### Statistical analysis

Correlation analyses of DNA methylation levels and gene expression, DNA methylation levels and EMT, gene expression, and EMT were assessed using Spearman’s correlation coefficient test by Matlab^®^ R2012a, statistics toolbox version 8.0 (MathWorks; Natick, MA, USA).

### Quantitative **reverse transcription-**PCR

Total RNA was extracted by RNeasy Mini Kit (Qiagen). Two hundred and fifty nanograms of RNA was reverse transcribed using RT^2^ First Strand Kit (Qiagen) and mixed with SYBR green master mix (Qiagen) for qPCR. Primers are listed in Supplementary Table 2. Normalized mRNA expression levels of each gene are presented either as average 2^−ΔCt^ or fold change 2^−ΔΔCt^.

### Western blotting and immunofluorescence staining

For western blotting, primary antibodies used include: anti-GRHL2 (HPA004820) from Sigma-Aldrich; anti-E-cadherin (610182) from BD Transduction Laboratories; anti-ZEB1 (3396) from Cell Signaling Technology; anti-vimentin (M7020) from Dako; anti-β-actin (A1978) from Sigma-Aldrich; anti-H3K27me3 (ab6002) and anti-H3 (ab24834) from Abcam; anti-H3Ac (06-599) from Millipore. Secondary antibodies from Li-COR Biosciences were used: IRDye 800CW goat anti-mouse/rabbit (926-32210, 926-32211) and IRDye 680LT goat anti-mouse/rabbit (926-68020, 926-68021). Blots were scanned using the Odyssey Infrared Imaging System (Li-COR). Full blots are shown in Supplementary Fig. 15. For immunofluorescence staining, primary antibodies used include anti-GRHL2 (HPA004820, Sigma-Aldrich) and anti-E-cadherin (610182, BD). Alexa Fluor 488-conjugated anti-rabbit (A11034) and Alexa Fluor 594-conjugated anti-mouse (A11032) from Invitrogen were used as secondary antibodies. Coverslips were mounted using Vectashield mounting medium with DAPI (4′,6-diamidino-2-phenylindole) (H-1200).

### Reporting summary

Further information on research design is available in the Nature Research Reporting Summary linked to this article.

## Supplementary information

Reporting Summary

Supplementary Data 1-10

Supplementary Information

Description of Additional Supplementary Items

## Data Availability

The DNA methylation, histone ChIP-seq, and gene expression data used in this study have been deposited on Gene Expression Omnibus (GEO) with accession number GSE118408.
