# Supplementary material for: The role of GRHL2 and epigenetic remodeling in epithelial–mesenchymal plasticity in ovarian cancer cells
Source: Commun Biol. 2019 Jul 24;2:272. doi: 10.1038/s42003-019-0506-3 (PMC6656769; doi:10.1038/s42003-019-0506-3)
Supplement: Supplementary file 1 — Reporting Summary [file 42003_2019_506_MOESM1_ESM.pdf]

nature research

Corresponding author(s): Huang RY, COMMSBIO-18-1142-B

Last updated by author(s): May 24, 2019

Reporting Summary

Nature Research wishes to improve the reproducibility of the work that we publish. This form provides structure for consistency and transparency in reporting. For further information on Nature Research policies, see Authors & Referees and the Editorial Policy Checklist.

Statistics

For all statistical analyses, confirm that the following items are present in the figure legend, table legend, main text, or Methods section.

n/a	Confirmed	
☐	☒	The exact sample size (n) for each experimental group/condition, given as a discrete number and unit of measurement
☐	☒	A statement on whether measurements were taken from distinct samples or whether the same sample was measured repeatedly
☐	☒	The statistical test(s) used AND whether they are one- or two-sided<br>*Only common tests should be described solely by name; describe more complex techniques in the Methods section.*
☐	☒	A description of all covariates tested
☐	☒	A description of any assumptions or corrections, such as tests of normality and adjustment for multiple comparisons
☐	☒	A full description of the statistical parameters including central tendency (e.g. means) or other basic estimates (e.g. regression coefficient) AND variation (e.g. standard deviation) or associated estimates of uncertainty (e.g. confidence intervals)
☐	☒	For null hypothesis testing, the test statistic (e.g. F, t, r) with confidence intervals, effect sizes, degrees of freedom and P value noted<br>*Give P values as exact values whenever suitable.*
☒	☐	For Bayesian analysis, information on the choice of priors and Markov chain Monte Carlo settings
☒	☐	For hierarchical and complex designs, identification of the appropriate level for tests and full reporting of outcomes
☒	☐	Estimates of effect sizes (e.g. Cohen's d, Pearson's r), indicating how they were calculated

Our web collection on statistics for biologists contains articles on many of the points above.

Software and code

Policy information about availability of computer code

Data collection	No software was used.
Data analysis	Matlab 9.1.0.441655 (R2016b).<br>homer version 4.7.2<br>R 3.1.2, bioconductor 2.6, ChAMP 1.2.8, MethylAid 1.1.0<br>Novoalign v3.02.06<br>phantompeakqualtools v1.1<br>samtools v0.1.19<br>bedtools 2.25.0<br>MACS2 v2.1.0.20150731

For manuscripts utilizing custom algorithms or software that are central to the research but not yet described in published literature, software must be made available to editors/reviewers. We strongly encourage code deposition in a community repository (e.g. GitHub). See the Nature Research guidelines for submitting code & software for further information.

Data

Policy information about availability of data

All manuscripts must include a data availability statement. This statement should provide the following information, where applicable:

- Accession codes, unique identifiers, or web links for publicly available datasets
- A list of figures that have associated raw data
- A description of any restrictions on data availability

deposited in GEO, accession: GSE118408

Field-specific reporting

Please select the one below that is the best fit for your research. If you are not sure, read the appropriate sections before making your selection.

☒ Life sciences ☐ Behavioural & social sciences ☐ Ecological, evolutionary & environmental sciences

For a reference copy of the document with all sections, see nature.com/documents/nr-reporting-summary-flat.pdf

Life sciences study design

All studies must disclose on these points even when the disclosure is negative.

Sample size	No sample size calculation was performed. the study investigated a list of cell lines available that represents the EMT spectrum.
Data exclusions	A cell line OV90 of poor quality (failed quality check) have been removed as stated in the manuscript methods and materials section.
Replication	For methylation, gene expression microarray, and RNA-seq, 2 replicates for each cell line were used. For drug-treatment/western triplicates were used.
Randomization	Category allocation was based on Epithelial-mesenchymal transition score.
Blinding	Blinding was not relevant to our study as we were using cell line models.

Reporting for specific materials, systems and methods

We require information from authors about some types of materials, experimental systems and methods used in many studies. Here, indicate whether each material, system or method listed is relevant to your study. If you are not sure if a list item applies to your research, read the appropriate section before selecting a response.

Materials & experimental systems

n/a	Involved in the study	
☐	☒	Antibodies
☐	☒	Eukaryotic cell lines
☒	☐	Palaeontology
☒	☐	Animals and other organisms
☒	☐	Human research participants
☒	☐	Clinical data

Methods

n/a	Involved in the study	
☐	☒	ChIP-seq
☒	☐	Flow cytometry
☒	☐	MRI-based neuroimaging

Antibodies

Antibodies used	Antibodies used for ChIP-seq: rabbit IgG (sc-2027, Santa Cruz), anti-H3K4me1 (ab8895, Abcam), anti-H3K4me3 (CS-003-100, Diagenode), anti-H3K27me3 (07-449, Millipore), anti-H3K27ac (ab4729, Abcam) and anti-H3K9me3 (ab8898, Abcam).<br>Antibodies used for western blotting: anti-ZEB1 (3396, Cell Signaling Technology), anti-Vimentin (M7020, Dako), anti-β-actin (A1978, Sigma-Aldrich), anti-H3K27me3 (ab6002) and anti-H3 (ab24834) from Abcam, anti-H3Ac (06-599, Millipore), IRDye 800CW goat anti-mouse/rabbit (926-32210, 926-32211) and IRDye 680LT goat anti-mouse/rabbit (926-68020, 926-68021) from Li-COR Biosciences.<br>Antibodies used for immunofluorescence staining: anti-GRHL2 (HPA004820, Sigma-Aldrich), anti-E-cadherin (610182, BD), Alexa Fluor 488-conjugated anti-rabbit (A11034) and Alexa Fluor 594-conjugated anti-mouse (A11032) from Invitrogen.
Validation	Based on manufacturers' validation results.

Eukaryotic cell lines

Policy information about cell lines

Cell line source(s)	Provided in Supplementary Table 1
Authentication	Authenticated by STR analysis
Mycoplasma contamination	Tested mycoplasma-negative or borderline using MycoAlert™ Mycoplasma Detection Kit
Commonly misidentified lines (See ICLAC register)	CH1-may be contaminated with PA1, also an ovarian cancer cell line. Hence it was not omitted.

ChIP-seq

Data deposition

☒ Confirm that both raw and final processed data have been deposited in a public database such as GEO.

☒ Confirm that you have deposited or provided access to graph files (e.g. BED files) for the called peaks.

Data access links<br>*May remain private before publication.*	https://www.ncbi.nlm.nih.gov/geo/query/acc.cgi?acc=GSE118408<br>token: qnslsgiuhhcjlqd
Files in database submission	GSM3319947 Gene expression of A2780 rep1<br>GSM3319948 Gene expression of A2780 rep2<br>GSM3319949 Gene expression of CAOV3 rep1<br>GSM3319950 Gene expression of CAOV3 rep2<br>GSM3319951 Gene expression of CH1 rep1<br>GSM3319952 Gene expression of CH1 rep2<br>GSM3319953 Gene expression of COLO720E rep1<br>GSM3319954 Gene expression of COLO720E rep2<br>GSM3319955 Gene expression of DOV13 rep1<br>GSM3319956 Gene expression of DOV13 rep2<br>GSM3319957 Gene expression of EFO21 rep1<br>GSM3319958 Gene expression of EFO21 rep2<br>GSM3319959 Gene expression of FUOV1 rep1<br>GSM3319960 Gene expression of FUOV1 rep2<br>GSM3319961 Gene expression of HEY rep1<br>GSM3319962 Gene expression of HEY rep2<br>GSM3319963 Gene expression of HEYC2 rep1<br>GSM3319964 Gene expression of HEYC2 rep2<br>GSM3319965 Gene expression of JHOS2 rep1<br>GSM3319966 Gene expression of JHOS2 rep2<br>GSM3319967 Gene expression of JHOS3 rep1<br>GSM3319968 Gene expression of JHOS3 rep2<br>GSM3319969 Gene expression of JHOS4 rep1<br>GSM3319970 Gene expression of JHSO4 rep2<br>GSM3319971 Gene expression of OAW28 rep1<br>GSM3319972 Gene expression of OAW28 rep2<br>GSM3319973 Gene expression of OAW42 rep1<br>GSM3319974 Gene expression of OAW42 rep2<br>GSM3319975 Gene expression of OV17R rep1<br>GSM3319976 Gene expression of OV17R rep2<br>GSM3319977 Gene expression of OV56 rep1<br>GSM3319978 Gene expression of OV56 rep2<br>GSM3319979 Gene expression of OV7 rep1<br>GSM3319980 Gene expression of OV7 rep2<br>GSM3319981 Gene expression of OVCA420 rep1<br>GSM3319982 Gene expression of OVCA420 rep2<br>GSM3319983 Gene expression of OVCA429 rep1<br>GSM3319984 Gene expression of OVCA429 rep2<br>GSM3319985 Gene expression of OVCA432 rep1<br>GSM3319986 Gene expression of OVCA432 rep2<br>GSM3319987 Gene expression of OVCA433 rep1<br>GSM3319988 Gene expression of OVCA433 rep2<br>GSM3319989 Gene expression of OVCAR2 rep1<br>GSM3319990 Gene expression of OVCAR2 rep2<br>GSM3319991 Gene expression of OVCAR3 rep1<br>GSM3319992 Gene expression of OVCAR3 rep2<br>GSM3319993 Gene expression of OVCAR5 rep1<br>GSM3319994 Gene expression of OVCAR5 rep2<br>GSM3319995 Gene expression of PEO1 rep1<br>GSM3319996 Gene expression of PEO1 rep2<br>GSM3319997 Gene expression of SKOV3 rep1<br>GSM3319998 Gene expression of SKOV3 rep2<br>GSM3319999 Gene expression of TOV112D rep1<br>GSM3320000 Gene expression of TOV112D rep2<br>GSM3320001 Gene expression of TYKNU rep1<br>GSM3320002 Gene expression of TYKNU rep2<br>GSM3320003 Gene expression of UWB1.289 rep1<br>GSM3320004 Gene expression of UWB1.289 rep2<br>GSM3327985 Methylation profile of A2780, replicate 1<br>GSM3327986 Methylation profile of A2780, replicate 2<br>GSM3327987 Methylation profile of CAOV3, replicate 1<br>GSM3327988 Methylation profile of CAOV3, replicate 2

GSM3327989 Methylation profile of CH1, replicate 1
GSM3327990 Methylation profile of CH1, replicate 2
GSM3327991 Methylation profile of COLO720E, replicate 1
GSM3327992 Methylation profile of COLO720E, replicate 2
GSM3327993 Methylation profile of DOV13, replicate 1
GSM3327994 Methylation profile of DOV13, replicate 2
GSM3327995 Methylation profile of EFO21, replicate 1
GSM3327996 Methylation profile of EFO21, replicate 2
GSM3327997 Methylation profile of FUOV1, replicate 1
GSM3327998 Methylation profile of FUOV1, replicate 2
GSM3327999 Methylation profile of HEY, replicate 1
GSM3328000 Methylation profile of HEY, replicate 2
GSM3328001 Methylation profile of HEYA8, replicate 1
GSM3328002 Methylation profile of HEYA8, replicate 2
GSM3328003 Methylation profile of HEYC2, replicate 1
GSM3328004 Methylation profile of HEYC2, replicate 2
GSM3328005 Methylation profile of JHOS2, replicate 1
GSM3328006 Methylation profile of JHOS2, replicate 2
GSM3328007 Methylation profile of JHOS3, replicate 1
GSM3328008 Methylation profile of JHOS3, replicate 2
GSM3328009 Methylation profile of JHOS4, replicate 1
GSM3328010 Methylation profile of JHOS4, replicate 2
GSM3328011 Methylation profile of OAW28, replicate 1
GSM3328012 Methylation profile of OAW28, replicate 2
GSM3328013 Methylation profile of OAW42, replicate 1
GSM3328014 Methylation profile of OAW42, replicate 2
GSM3328015 Methylation profile of OV17R, replicate 1
GSM3328016 Methylation profile of OV17R, replicate 2
GSM3328017 Methylation profile of OV56, replicate 1
GSM3328018 Methylation profile of OV56, replicate 2
GSM3328019 Methylation profile of OV7, replicate 1
GSM3328020 Methylation profile of OV7, replicate 2
GSM3328021 Methylation profile of OVCA420, replicate 1
GSM3328022 Methylation profile of OVCA420, replicate 2
GSM3328023 Methylation profile of OVCA429, replicate 1
GSM3328024 Methylation profile of OVCA429, replicate 2
GSM3328025 Methylation profile of OVCA432, replicate 1
GSM3328026 Methylation profile of OVCA432, replicate 2
GSM3328027 Methylation profile of OVCA433, replicate 1
GSM3328028 Methylation profile of OVCA433, replicate 2
GSM3328029 Methylation profile of OVCAR2, replicate 1
GSM3328030 Methylation profile of OVCAR2, replicate 2
GSM3328031 Methylation profile of OVCAR3, replicate 1
GSM3328032 Methylation profile of OVCAR3, replicate 2
GSM3328033 Methylation profile of OVCAR5, replicate 1
GSM3328034 Methylation profile of OVCAR5, replicate 2
GSM3328035 Methylation profile of PEO1, replicate 1
GSM3328036 Methylation profile of PEO1, replicate 2
GSM3328037 Methylation profile of SKOV3, replicate 1
GSM3328038 Methylation profile of SKOV3, replicate 2
GSM3328039 Methylation profile of TOV112D, replicate 1
GSM3328040 Methylation profile of TOV112D, replicate 2
GSM3328041 Methylation profile of TYKNu, replicate 1
GSM3328042 Methylation profile of TYKNu, replicate 2
GSM3328043 Methylation profile of UWB1.289, replicate 1
GSM3328044 Methylation profile of UWB1.289, replicate 2
GSM3328045 Methylation profile of OVCA429 shGRHL2, replicate 1
GSM3328046 Methylation profile of OVCA429 shGRHL2, replicate 2
GSM3328047 Methylation profile of OVCA429 shNon control, replicate 1
GSM3328048 Methylation profile of OVCA429 shNon control, replicate 2
GSM3328049 PEO1 input DNA
GSM3328050 PEO1 with H3K4me3 ChIP
GSM3328051 PEO1 with H3K4me1 ChIP
GSM3328052 PEO1 with H3K27ac ChIP
GSM3328053 PEO1 with H3K27me3 ChIP
GSM3328054 PEO1 with H3K9me3 ChIP
GSM3328055 OVCA429 input DNA
GSM3328056 OVCA429 with H3K4me3 ChIP
GSM3328057 OVCA429 with H3K4me1 ChIP
GSM3328058 OVCA429 with H3K27ac ChIP
GSM3328059 OVCA429 with H3K27me3 ChIP
GSM3328060 OVCA429 with H3K9me3 ChIP
GSM3328061 SKOV3 input DNA
GSM3328062 SKOV3 with H3K4me3 ChIP
GSM3328063 SKOV3 with H3K4me1 ChIP

GSM3328064 SKOV3 with H3K27ac ChIP
GSM3328065 SKOV3 with H3K27me3 ChIP
GSM3328066 SKOV3 with H3K9me3 ChIP
GSM3328067 HEYA8 input DNA
GSM3328068 HEYA8 with H3K4me3 ChIP
GSM3328069 HEYA8 with H3K4me1 ChIP
GSM3328070 HEYA8 with H3K27ac ChIP
GSM3328071 HEYA8 with H3K27me3 ChIP
GSM3328072 HEYA8 with H3K9me3 ChIP
GSM3328073 OVCA429shLuc input DNA
GSM3328074 OVCA429shLuc with H3K4me3 ChIP
GSM3328075 OVCA429shLuc with H3K4me1 ChIP
GSM3328076 OVCA429shLuc with H3K27ac ChIP
GSM3328077 OVCA429shLuc with H3K27me3 ChIP
GSM3328078 OVCA429shLuc with H3K9me3 ChIP
GSM3328079 OVCA429shGRHL2 input DNA
GSM3328080 OVCA429shGRHL2 with H3K4me3 ChIP
GSM3328081 OVCA429shGRHL2 with H3K4me1 ChIP
GSM3328082 OVCA429shGRHL2 with H3K27ac ChIP
GSM3328083 OVCA429shGRHL2 with H3K27me3 ChIP
GSM3328084 OVCA429shGRHL2 with H3K9me3 ChIP
GSM3328085 OVCA429 with shLuciferase rep1
GSM3328086 OVCA429 with shLuciferase rep2
GSM3328087 OVCA429 with shLuciferase rep3
GSM3328088 OVCA429 with shLuciferase rep4
GSM3328089 OVCA429 with shGRHL2 rep1
GSM3328090 OVCA429 with shGRHL2 rep2
GSM3328091 OVCA429 with shGRHL2 rep3
GSM3328092 OVCA429 with shGRHL2 rep4

Genome browser session
(e.g. UCSC)

https://genome-asia.ucsc.edu/cgi-bin/hgTracks?hgS_doLoadUrl=submit&hgS_loadUrlName=http://csibio.nus.edu.sg/CSIOVDB/tmp/VYetal.txt

Methodology

Replicates

No replicate for ChIP-seq experiment

Sequencing depth

Single-end
Sample Total read Unique Mapped Read
HeyA8_H3K27ac 47842610 45945994
HeyA8_H3K27me3 42335504 38429322
HeyA8_H3K4me1 53046412 50868697
HeyA8_H3K4me3 57825334 53387969
HeyA8_H3K9me3 48195895 36162229
HeyA8_Input 42589623 39242016
OVCA429 shGRHL2_12_H3K27ac 47814401 45698153
OVCA429 shGRHL2_12_H3K27me3 50546729 45758558
OVCA429 shGRHL2_12_H3K4me1 52237685 49914076
OVCA429 shGRHL2_12_H3K4me3 47339468 43602727
OVCA429 shGRHL2_12_H3K9me3 40060637 31689252
OVCA429 shGRHL2_12_Input 34020999 31162410
OVCA429 shLuc_H3K27ac 47145506 45070299
OVCA429 shLuc_H3K27me3 46078739 41775257
OVCA429 shLuc_H3K4me1 48485617 46102838
OVCA429 shLuc_H3K4me3 45449067 42122732
OVCA429 shLuc_H3K9me3 43850346 35450648
OVCA429 shLuc_Input 52940484 48553529
OVCA429_H3K27ac 62959144 59806042
OVCA429_H3K27me3 54749548 49803827
OVCA429_H3K4me1 48092565 45477678
OVCA429_H3K4me3 55064154 50341752
OVCA429_H3K9me3 45969224 35995352
OVCA429_Input 39412580 35882338
PEO1_H3K27ac 56139290 53374216
PEO1_H3K27me3 51807251 46511281
PEO1_H3K4me1 40379391 38322260
PEO1_H3K4me3 45891843 42623386
PEO1_H3K9me3 60908677 48483852
PEO1_Input 59179834 54079658
Skov3_H3K27ac 45283022 33185117
Skov3_H3K27me3 56322804 50105581
Skov3_H3K4me1 21506343 20016074
Skov3_H3K4me3 48977666 45041084
Skov3_H3K9me3 49180425 40481524

	Skov3_Input 44516504 40708404
Antibodies	Antibodies used include rabbit IgG (sc-2027, Santa Cruz), anti-H3K4me1 (ab8895, Abcam), anti-H3K4me3 (CS-003-100, Diagenode), anti-H3K27me3 (07-449, Millipore), anti-H3K27ac (ab4729, Abcam) and anti-H3K9me3 (ab8898, Abcam)
Peak calling parameters	-p 1e-3 --to-large<br>subsequently IDR overlap between pseudo-replicates
Data quality	Quality check was performed according SPP IDR pipeline.
Software	homer version 4.7.2<br>R 3.1.2, bioconductor 2.6, ChAMP 1.2.8, MethylAid 1.1.0<br>Novoalign v3.02.06<br>phantompeakqualtools v1.1<br>samtools v0.1.19<br>bedtools 2.25.0<br>MACS2 v2.1.0.20150731
