# Supplementary material for: The role of GRHL2 and epigenetic remodeling in epithelial–mesenchymal plasticity in ovarian cancer cells
Source: Commun Biol. 2019 Jul 24;2:272. doi: 10.1038/s42003-019-0506-3 (PMC6656769; doi:10.1038/s42003-019-0506-3)
Supplement: Supplementary file 3 — Supplementary Information [file 42003_2019_506_MOESM3_ESM.pdf]

Supplementary Notes

EMT-correlated DMCs in different EMT signature gene sets.

The reproducibility of our results was tested using EMT signature gene sets defined by other studies. For this purpose, we obtained the hallmark EMT gene set from molecular signatures database (Msigdb v6.1)[1]. This hallmark EMT signature fully comprised Mes genes, with only 2 genes (*LAMC2*, *VIM*) that overlapped with the EMT signature we derived. Consistent with our finding of 4 out of 223 (1%) Mes genes that harbored EMT− DMC at TSS, only 6 out of 168 Mes genes (3.5%) in this gene set contained EMT− DMC at TSS (Supplementary Data 4). 21 out of 170 Mes genes had intragenic EMT+ DMCs whereas 4 out of 170 Mes genes contained intragenic EMT− DMCs. On the other hand, no hallmark gene set was available for epithelial genes. Hence, we selected the genes listed in Gene Ontology[2] epithelial differentiation gene set with gene expressions that significantly anti-correlate with EMT ($p < 0.05$). In this gene set, we found 20 over 45 Epi genes (44.4%) that harbored EMT+ DMCs at TSS, an observation similar to what we reported. 10 out of 46 Epi genes (21.7%) had EMT+ DMCs whereas 10 other Epi genes had EMT− DMCs in their gene bodies, respectively. In summary, using two different gene sets related to EMT, we obtained similar findings in OC cells, suggesting that Epi genes were more susceptible to DNA methylation-mediated regulation, as compared to Mes genes.

EMT-correlated DMCs in clinical tumor samples.

We tested if the observation of epithelial genes being more susceptible to DNA methylation could be reproduced in clinical samples of ovarian and other cancers. To this end, we checked whether the correlation between CpG methylation and EMT score (computed

from gene expression) of clinical samples were concordant with that of ovarian cancer cell lines. To perform the analyses, we obtained the methylation profiles (Illumina Infinium 450K array data) of ovarian and other cancers of the TCGA cohorts[3]. Only 9 samples in the TCGA ovarian cancer cohort had both 450K and RNA-seq data. Hence, we included additional ovarian tumor samples of the Duke cohort (GSE51820)[4] for analyses alongside. The CpG sites analysed were selected based on two criteria—1: EMT-correlated DMCs identified in ovarian cancer cell lines, 2: located at TSS of the EMT signature genes. Interestingly, several cancer types such as the breast and bladder cancer (BLCA) showed consistent EMT-methylation correlation at CpG sites near TSS of epithelial genes but only marginal or no correlation in mesenchymal genes (Supplementary Fig. 3). This suggested that our finding of epithelial genes being more susceptible to DNA methylation regulation could be applied not only to ovarian cancer cell lines but also to clinical samples of ovarian and other cancer types. Worth noting, the sarcoma-like mesenchymal tumors such as GBM and SKCM showed no correlation at the selected CpG sites.

Supplementary Figures

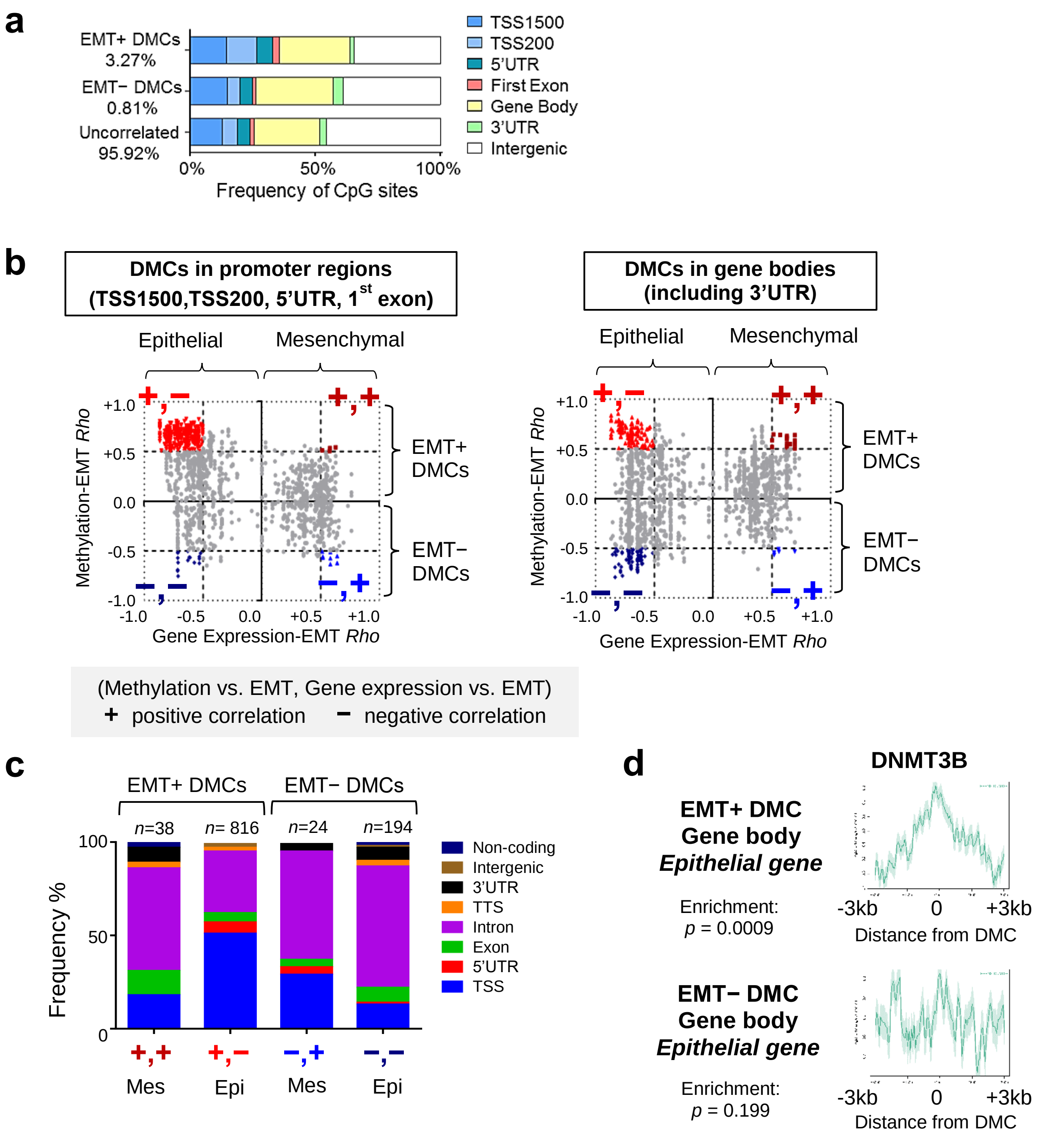

Supplementary Fig. 1. EMT-correlated DMCs.

a) Bar chart indicates the distribution of EMT+ DMCs, EMT− DMCs and non-EMT-correlated CpG sites in different genomic compartments. **b**) Scatter plots of genes with DMCs in promoter regions (left) and genes with DMCs in gene bodies (right) showing their respective CpG methylation-EMT correlation (*Rho*, *y*-axis) and gene expression-EMT correlation (*Rho*, *x*-axis). **c**) Bar chart indicates the genomic distribution of DMCs associated with Epi (Epithelial) or Mes (Mesenchymal) genes, based on correlation with EMT. **d**) Comparison of DNMT3B binding (ChIP-seq data of HEPG2 cells) between intragenic EMT+ DMCs and intragenic EMT− DMCs associated with Epithelial genes.

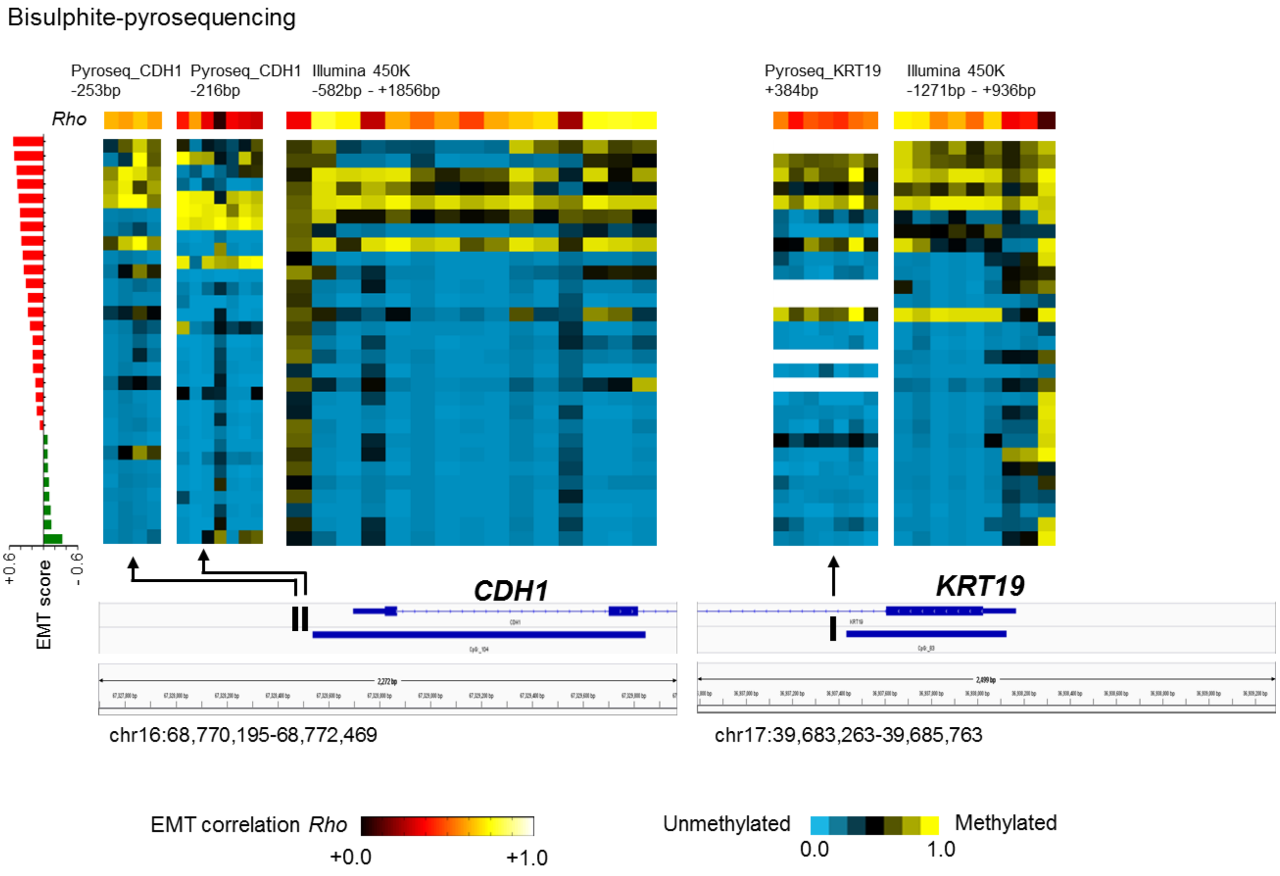

Supplementary Fig. 2. Validation of 450K array with bisulphite-pyrosequencing.
Heat maps comparing the results of Illumina's HumanMethylation 450K array with bisulphite-pyrosequencing: methylation status of CpG sites near the promoter regions of CDH1 and KRT19 in 29 OC cell lines with progressive EMT scores. Genome positions shown are in hg19.

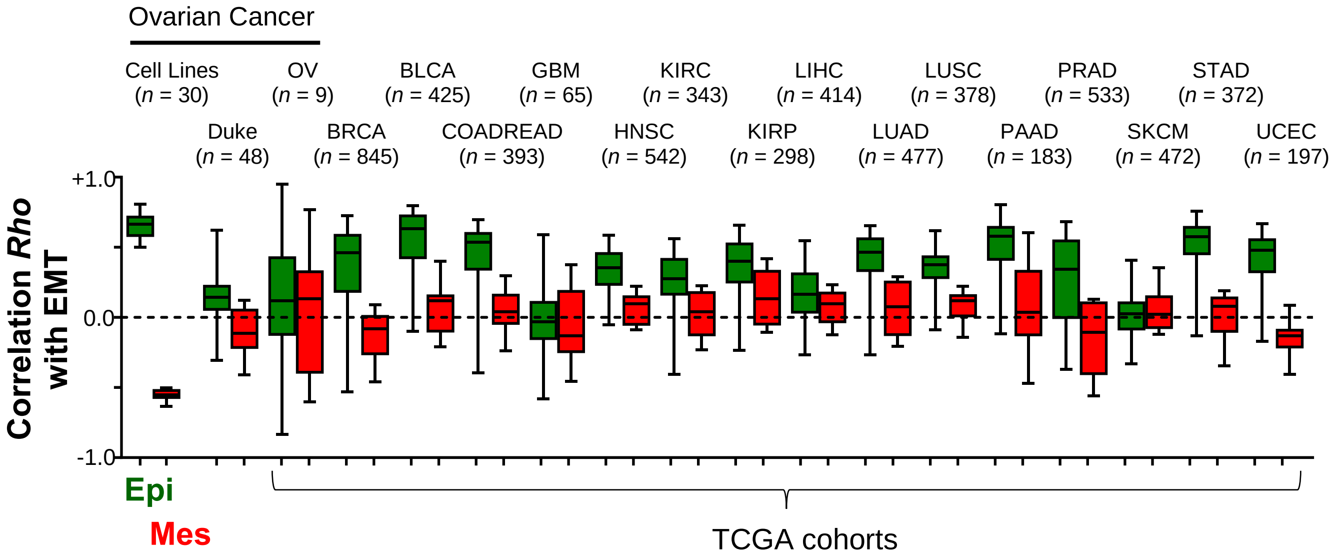

Supplementary Fig. 3. Comparison between EMT-correlated DMCs in ovarian cancer cell lines and clinical tumor samples.

Box plots showing Spearman correlation coefficient *Rho* between EMT score and DNA methylation levels (*y*-axis) of EMT-correlated DMCs at TSS of EMT signature genes in ovarian cancer cell lines, GSE51820 (Duke) ovarian cancer cohort[4], and different cancer samples in the TCGA cohorts[3] (*x*-axis). Box represents 1st, 2nd, and 3rd quantiles; whiskers are min and max values. (Epi) Epithelial genes are in green; (Mes) Mesenchymal genes are in red. Abbreviations: BLCA, bladder urothelial carcinoma; BRCA, breast invasive carcinoma; COADREAD, colorectal adenocarcinoma; GBM, glioblastoma; HNSC, head and neck squamous cell carcinoma; KIRC, kidney renal cell carcinoma; KIRP, kidney renal papillary cell carcinoma; LIHC, liver hepatocellular carcinoma; LUAD, lung adenocarcinoma; LUSC, lung squamous cell carcinoma; OV, ovarian serous cystadenocarcinoma; PAAD, pancreatic adenocarcinoma; PRAD, prostate adenocarcinoma; SKCM, skin cutaneous melanoma; STAD, stomach adenocarcinoma; UCEC, uterine corpus endometrial carcinoma.

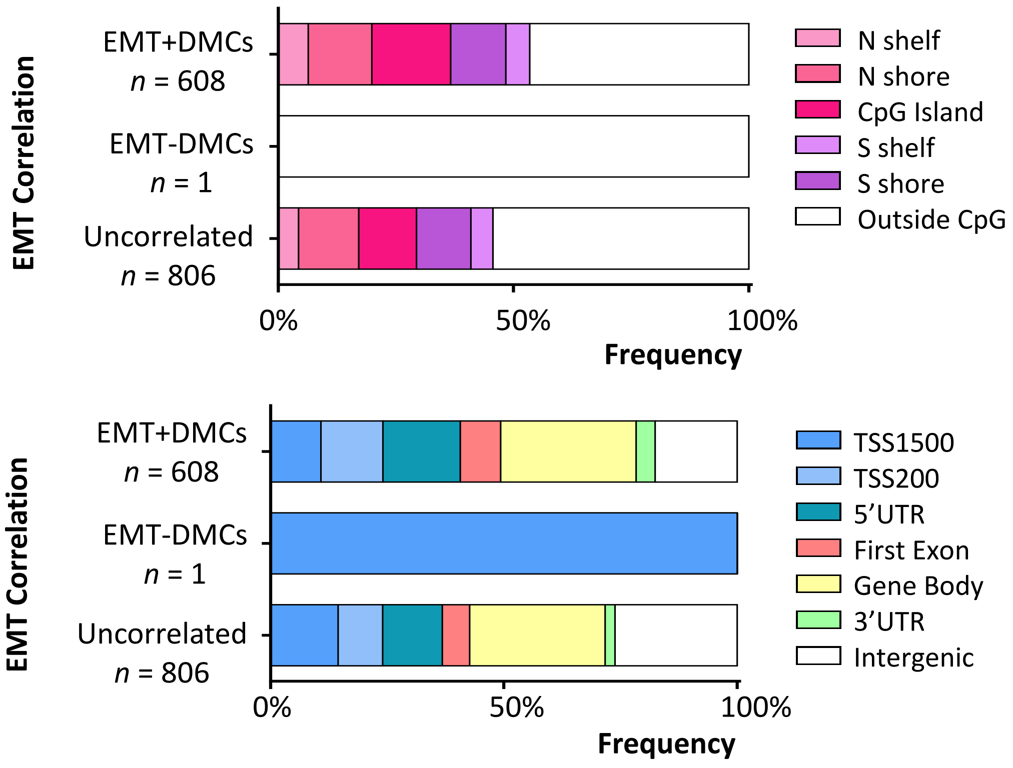

Supplementary Fig. 4. EMT+ DMCs within GRHL2 binding sites.

Bar charts indicate the genomic and CpG island distributions of EMT+ DMCs that co-occurred with GRHL2 binding sites

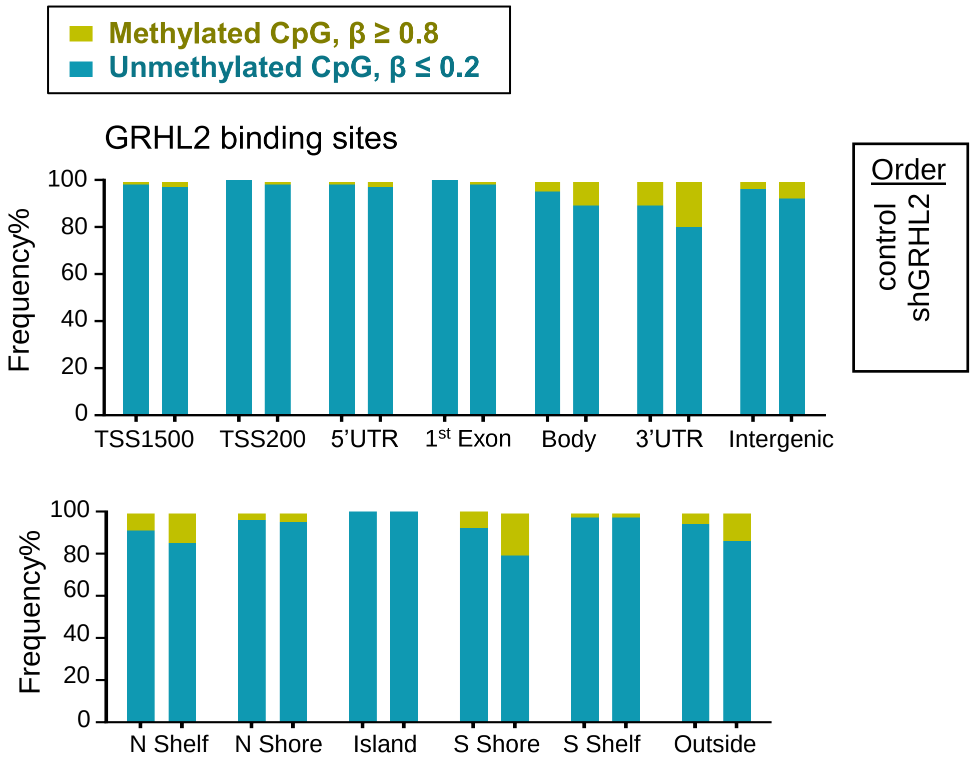

Supplementary Fig. 5. CpG methylation at GRHL2 binding sites in GRHL2-knockdown model.

Bar charts showing the frequency (%) of methylated ($\beta \geq 0.8$) and unmethylated ($\beta \leq 0.2$) CpG at GRHL2 binding sites of different genomic loci/compartments in control and GRHL2-knockdown OVCA429 cells.

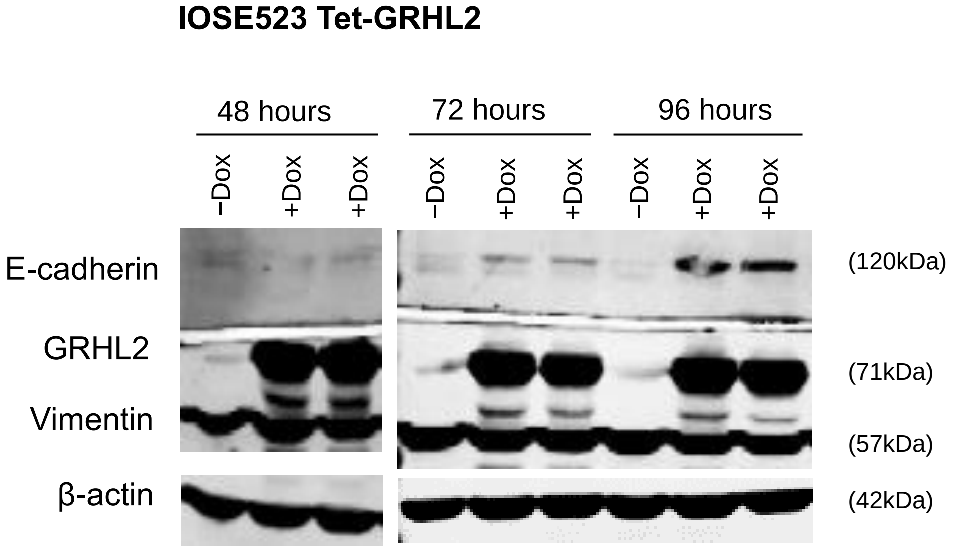

Supplementary Fig. 6. GRHL2 overexpression in IOSE523 Tet-GRHL2 resulted in upregulation of E-cadherin.

Western blots showing protein levels of E-cadherin, GRHL2, vimentin and β-actin in IOSE523 Tet-GRHL2 cells with/without doxycycline treatment (to induce GRHL2 expression) for 48, 72 or 96 hours.

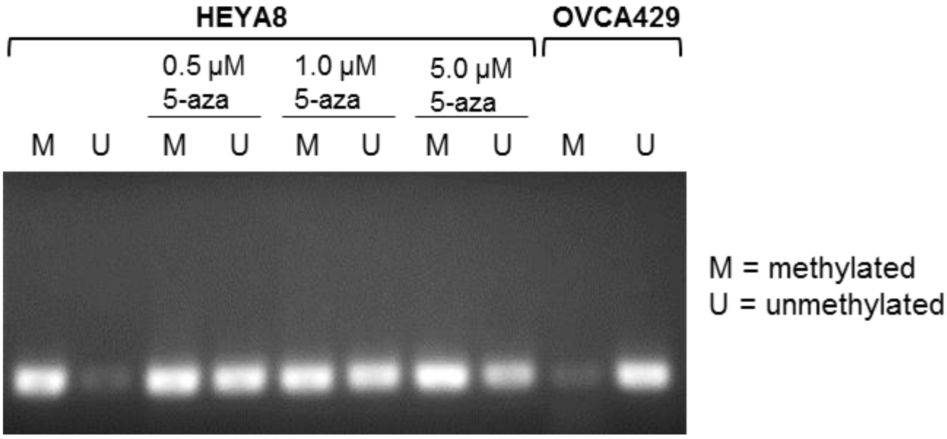

Supplementary Fig. 7. Effects of 5-azacitidine treatment on DNA methylation. Methylation-specific PCR results showing DNA methylation status of *CDH1* promoter in HEYA8 before and after 5-azacitidine treatment with different concentrations and in OVCA429. Primers used were based on previously reported study[5] (Supplementary Table 2).

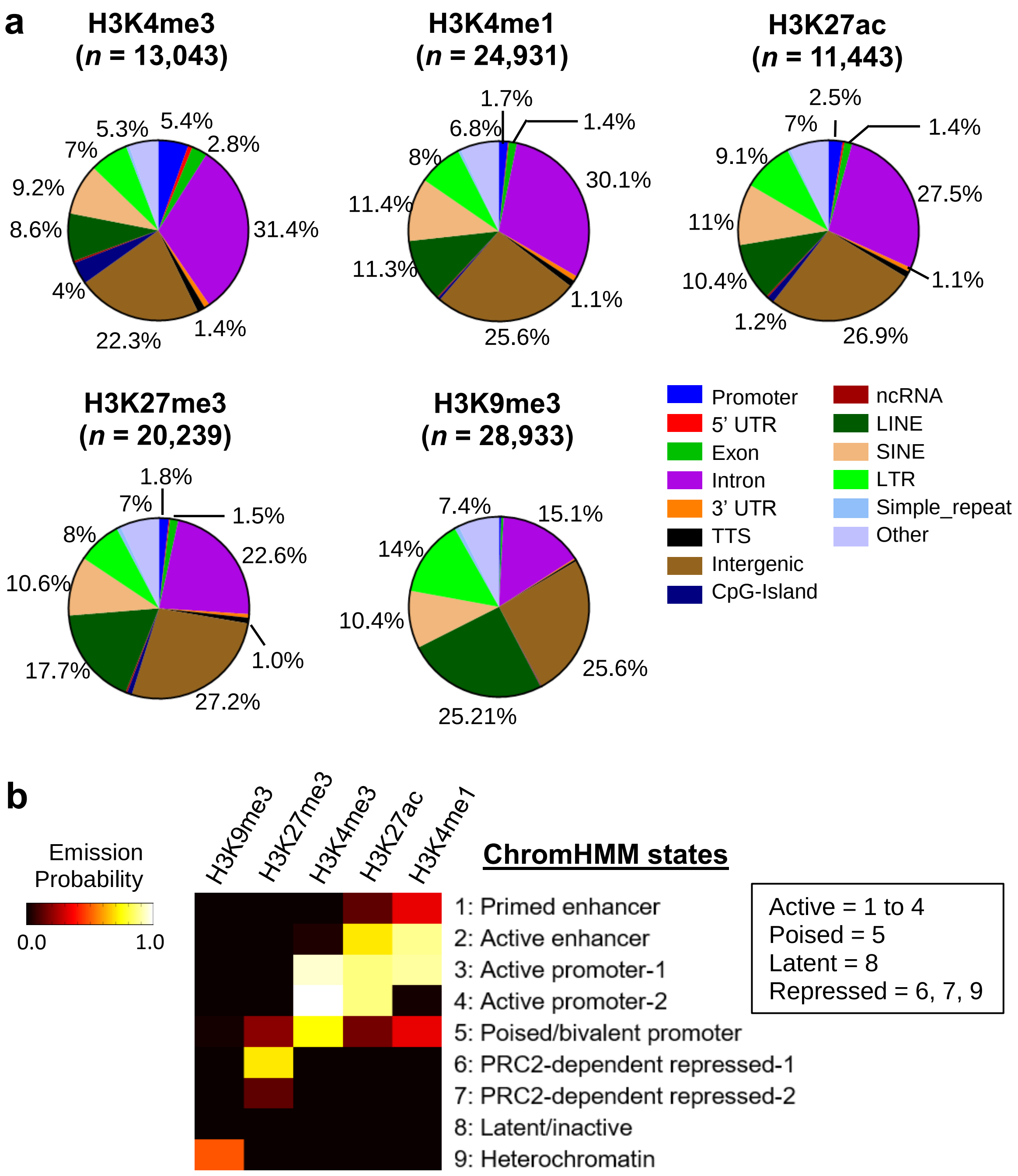

Supplementary Fig. 8. The distribution and combinations of histone H3 modification and the resulted ChromHMM states.

a) Pie charts portraying the distribution of different histone H3 marks (H3K4me3, H3K4me1, H3K27ac, H3K27me3, and H3K9me3) in various genomic compartments. **b**) Emission probability heatmap showing the nine ChromHMM states derived from different combinatorial levels of the five histone marks tested—H3K9me3, H3K27me3, H3K4me3, H3K27ac, and H3K4me1. States 1 to 4 are grouped as active while states 6, 7 and 9 are grouped as repressed.

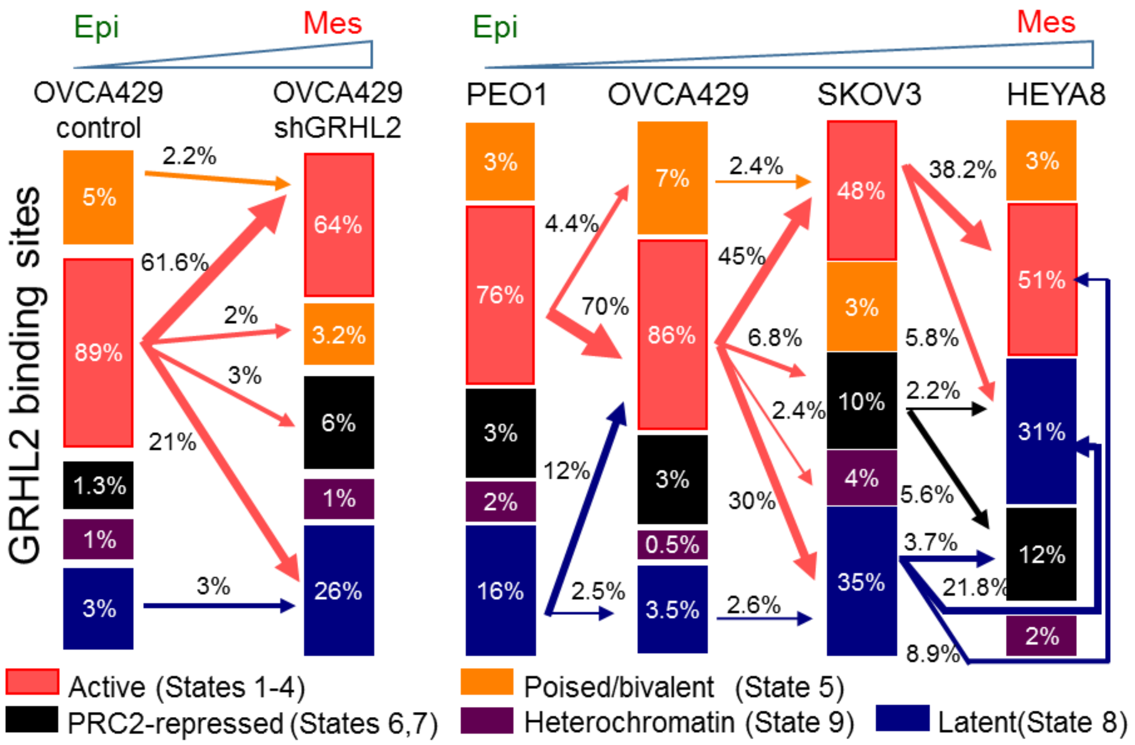

Supplementary Fig. 9. ChromHMM state changes at GRHL2 binding sites.
Diagrams showing the percentage of GRHL2 binding sites with different ChromHMM state transition in the GRHL2-knockdown EMT model (OVCA429 control vs. shGRHL2) and the four-cell-line EMT model (PEO1, OVCA429, SKOV3, HEYA8). Only transitions with percentage $\geq$ 2% are shown.

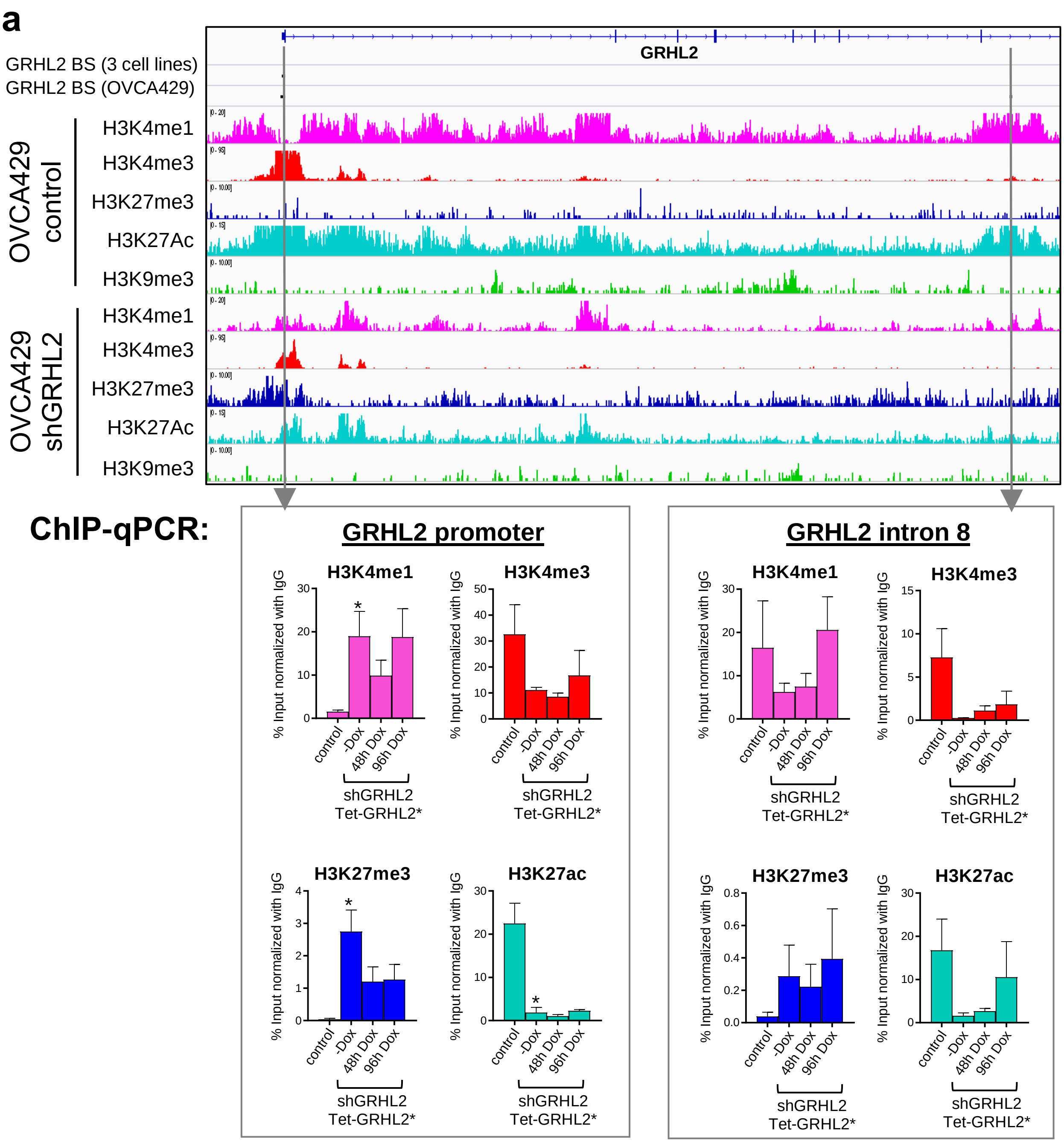

Supplementary Fig. 10. Validation of ChIP-seq by ChIP-qPCR in OVCA429 GRHL2-knockdown and Tet-GRHL2* rescue models.

Diagrams depict the ChIP-sequencing results (top panel) of H3K4me1, H3K4me3, H3K27me3, H3K27ac, and H3K9me3 in OVCA429 control and shGRHL2 cells, showing the loci of primers (grey arrows) used for ChIP-qPCR (bottom panel) of the same histone marks except for H3K9me3 in OVCA429 control and OVCA429 shGRHL2 Tet-GRHL2* cells with/without doxycycline-induced GRHL2 overexpression, at GRHL2 (**a**), CDH1 (**b**) and OVOL2 (**c**) genes. BS refers to binding site. Error bars = s.e.m.

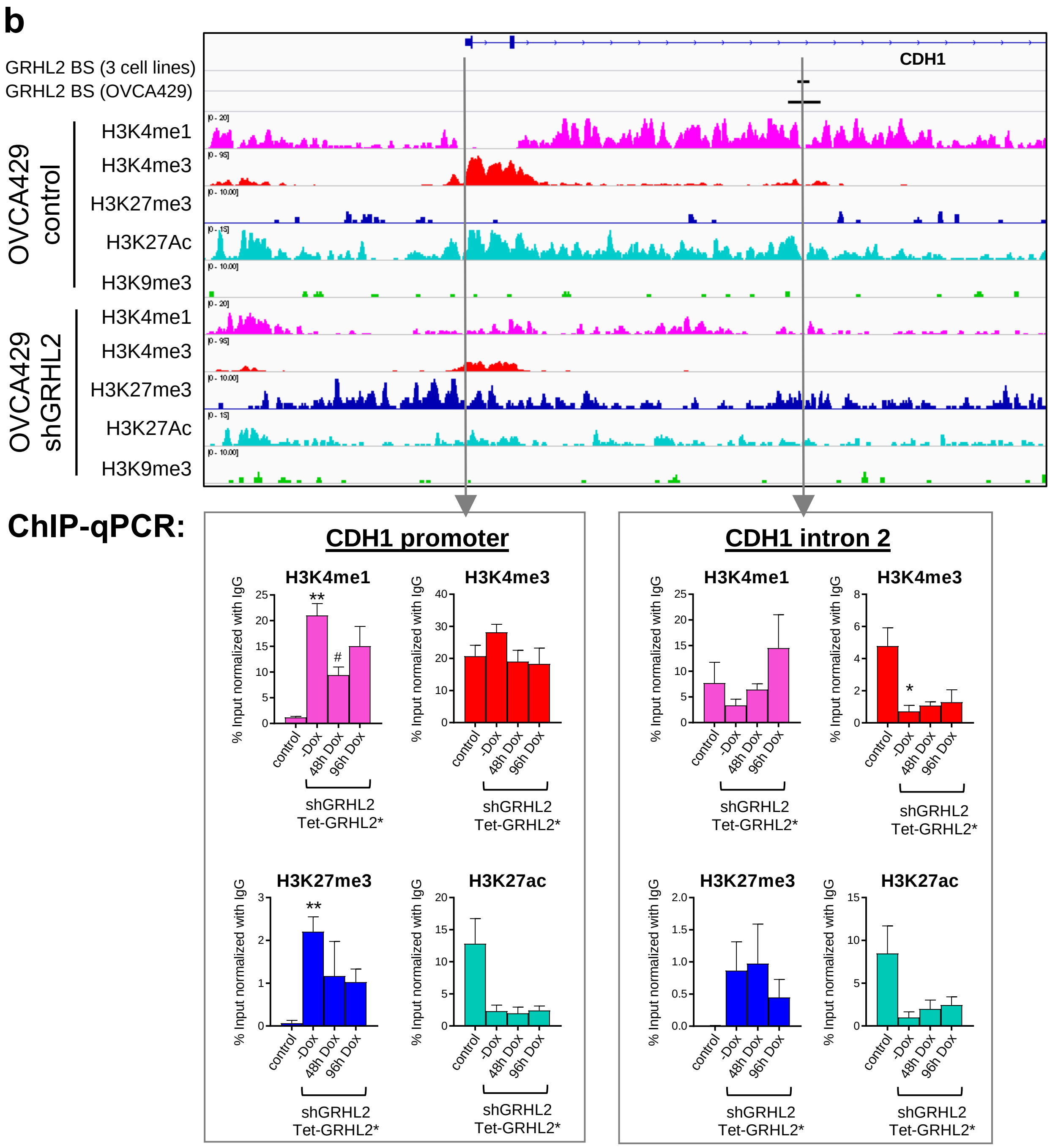
b
GRHL2 BS (3 cell lines)
GRHL2 BS (OVCA429)
CDH1
OVCA429 control
OVCA429 shGRHL2
H3K4me1
H3K4me3
H3K27me3
H3K27Ac
H3K9me3
ChIP-qPCR:
CDH1 promoter
CDH1 intron 2
H3K4me1
H3K4me3
H3K27me3
H3K27ac
% Input normalized with IgG
control
-Dox
48h Dox
96h Dox
shGRHL2
Tet-GRHL2*

c

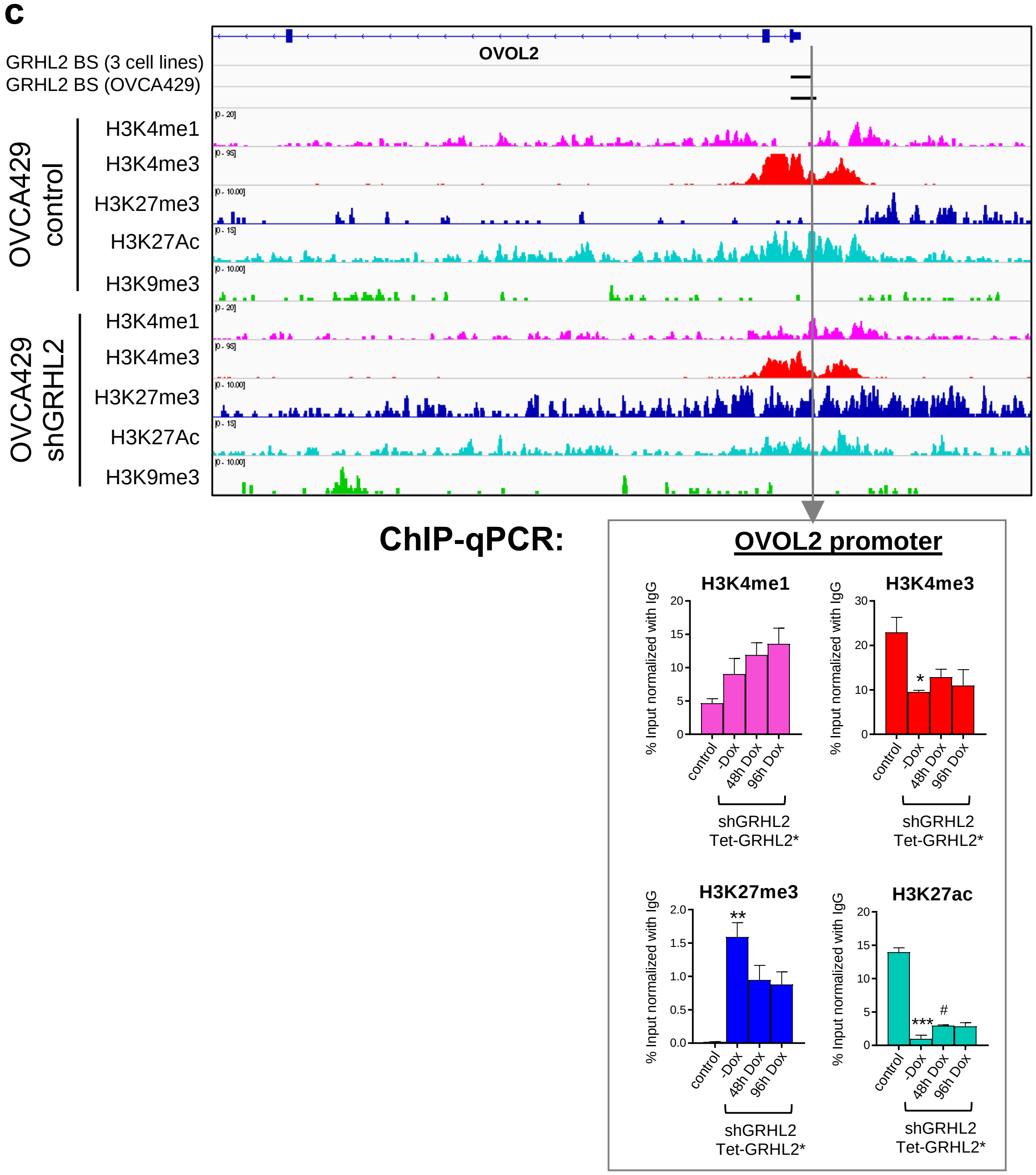

OVOL2
GRHL2 BS (3 cell lines)
GRHL2 BS (OVCA429)
OVCA429 control
H3K4me1
H3K4me3
H3K27me3
H3K27Ac
H3K9me3
OVCA429 shGRHL2
H3K4me1
H3K4me3
H3K27me3
H3K27Ac
H3K9me3
ChIP-qPCR:
OVOL2 promoter
H3K4me1
H3K4me3
H3K27me3
H3K27ac
% Input normalized with IgG
control
-Dox
48h Dox
96h Dox
shGRHL2
Tet-GRHL2*

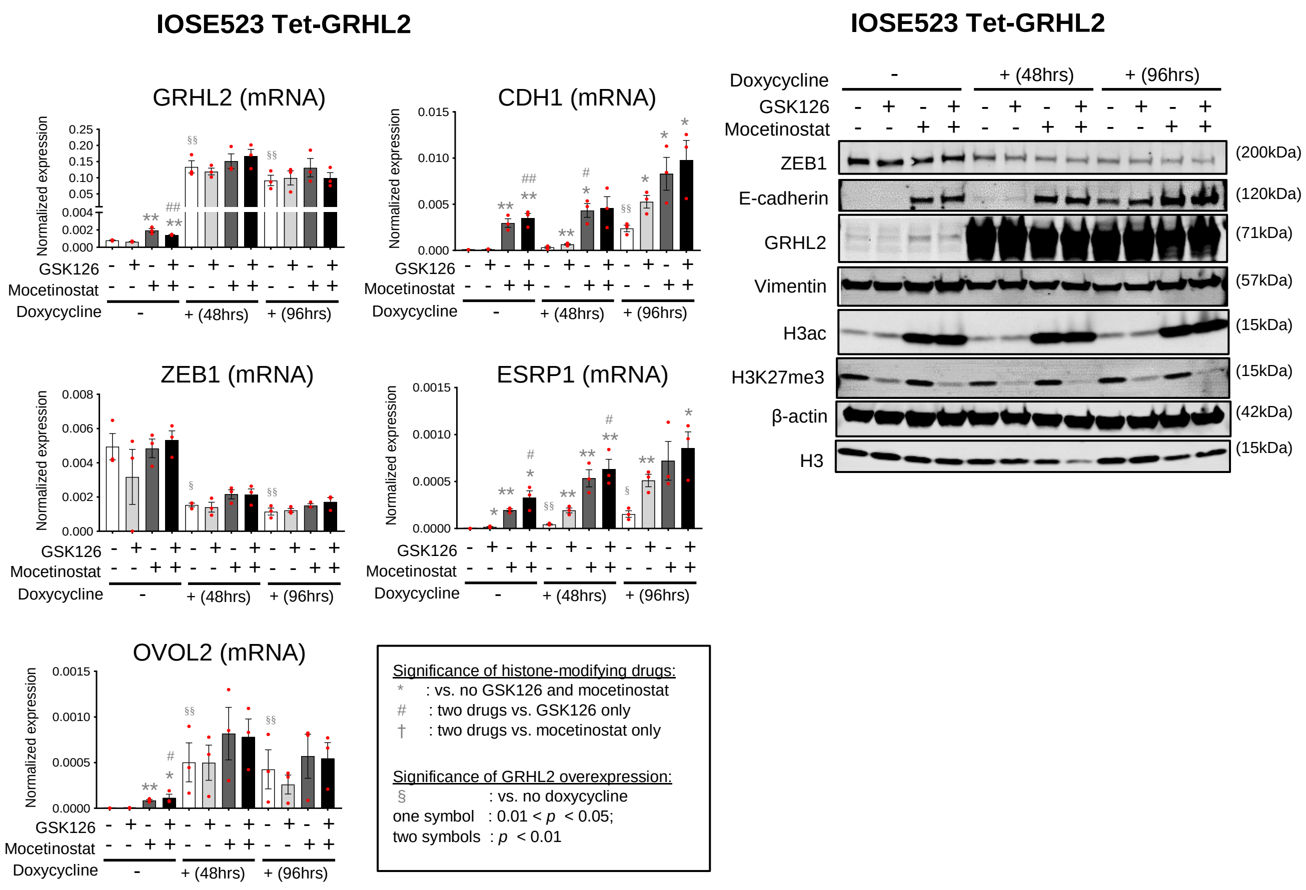

Supplementary Fig. 11. Effects of GSK126 and mocetinostat in combination with GRHL2 overexpression in IOSE523 Tet-GRHL2.

Bar graphs (left) depict the normalized mRNA expression of *GRHL2*, *CDH1*, *ZEB1*, *ESRP1* and *OVOL2* in IOSE523 Tet-GRHL2 cells with/without doxycycline treatment (to induce GRHL2 expression) for 48 or 96 hours, along with/without GSK126 (EZH2 inhibitor) and/or mocetinostat (HDAC inhibitor). Error bars = s.e.m. Statistical significance based on biological triplicates: * represents significance of histone drug-treated vs. no histone drug treatment; # represents significance of two drugs combined vs. GSK126 treatment only; † represents significance of two drugs combined vs. mocetinostat treatment only; § represents significance of doxycycline-treated vs. no treatment control. Western blots (right) of ZEB1, E-cadherin, GRHL2, vimentin, H3ac, H3K27me3, β-actin and total H3 in IOSE523 Tet-GRHL2 cells with/without doxycycline treatment (to induce GRHL2 expression) for 48 or 96 hours, with/without treatment of GSK126 and/or mocetinostat.

HEYA8 Tet-GRHL2:

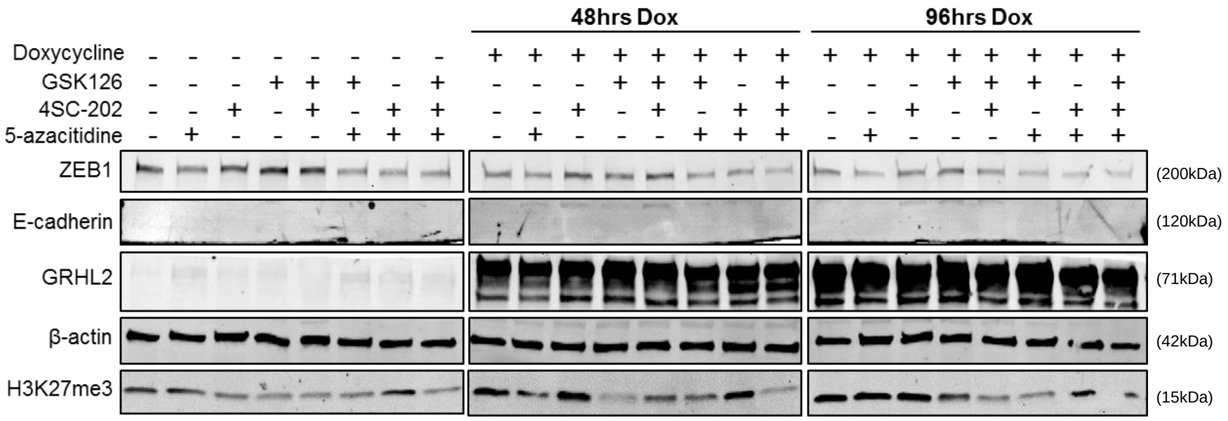

Supplementary Fig. 12. GRHL2 overexpression in combination with GSK126, 4SC-202 and/or 5-azacitidine.

Western blots showing protein levels of ZEB1, E-cadherin, GRHL2, β-actin, and H3K27me3 in HEYA8 Tet-GRHL2 cells with/without doxycycline (to induce GRHL2 expression) for 48 or 96 hours in combination with GSK126 (72 hours), 4SC-202 (48 hours) or 5-azacitidine (72 hours). No upregulation of E-cadherin was observed.

HEYA8 cells:

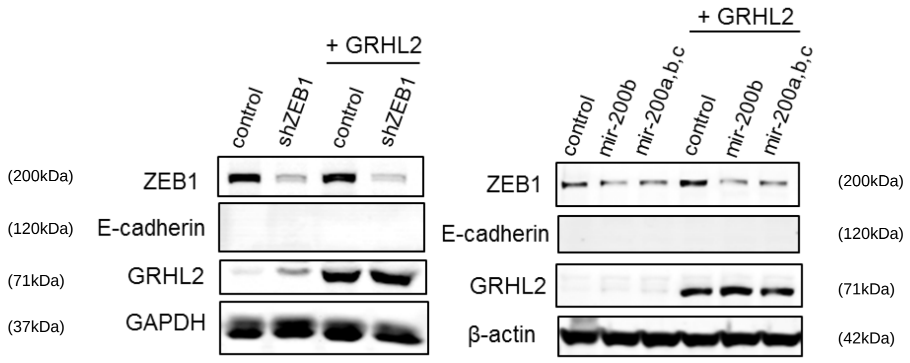

Supplementary Fig. 13. GRHL2 overexpression in combination with ZEB1 knockdown or mir-200 overexpression.

Western blots showing protein levels of ZEB1, E-cadherin, GRHL2, and GAPDH or β-actin in HEYA8 cells with/without GRHL2 overexpression in combination with ZEB1 knockdown (left panel) or with mir-200 overexpression (right panel). No upregulation of E-cadherin was observed.

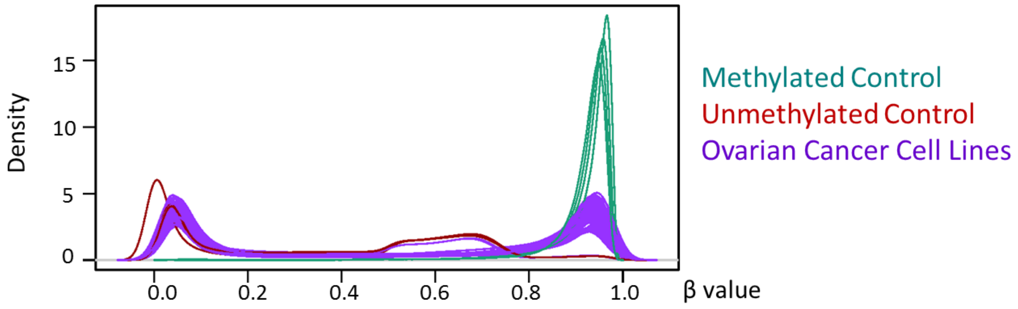

Supplementary Fig. 14. Beta-distribution of the β value normalization.
The density of CpG sites distributed based on β values in methylated control, unmethylated control and ovarian cancer cell lines tested.

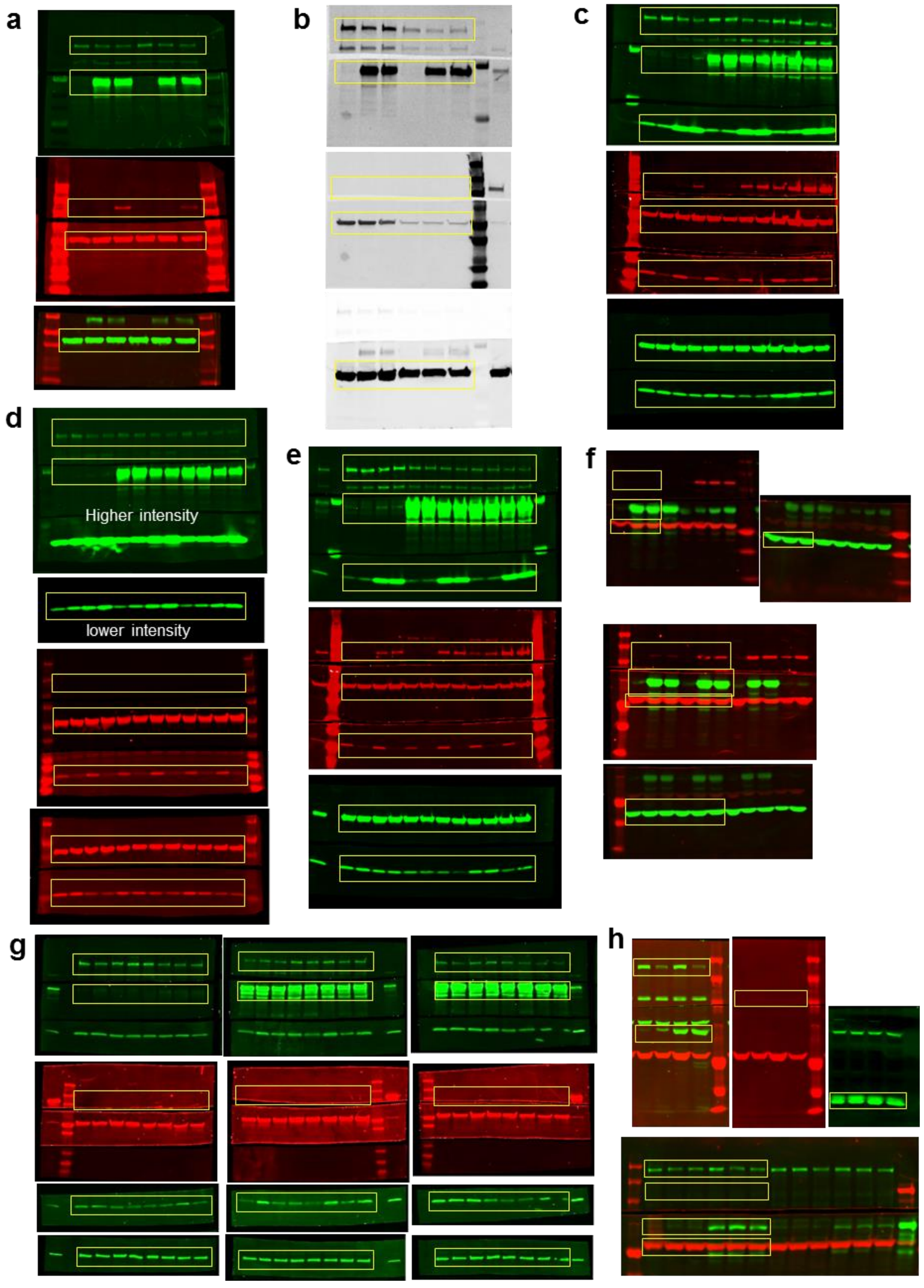

Supplementary Fig. 15. Full western blots

a) Original full western blots shown in Figure 3a. **b**) Original full western blots shown in Figure 3b. **c**) Original full western blots shown in Figure 6b. **d**) Original full western blots shown in Figure 6d. **e**) Original full western blots shown in Supplementary Figure 6. **f**) Original full western blots shown in Supplementary Figure 11. **g**) Original full western blots shown in Supplementary Figure 12. **h**) Original full western blots shown in Supplementary Figure 13.

Supplementary Tables

Supplementary Table 1. Cell lines used are listed with their respective EMT scores, usage in other studies, origins (based on Cellosaurus) and sources.

Cell Line	EMT Score (Ovarian Cancer)	Used in Domcke *et al*, 2013	Used in Beaufort *et al*, 2014	Used in Papp *et al*, 2018	Cellosaurus.v29 UniqueID	Cellosaurus.v29 Disease_Origin	Cellosaurus.v29 Sources	Cell Line Sources
PEO1	-0.335		✓		CVCL_2686	Ovarian cystadenocarcinoma	ECACC	ECACC
JHOS4	-0.139	✓		✓	CVCL_4649	High grade ovarian serous adenocarcinoma	RCB	RIKEN
OVCA420	-0.131				CVCL_3935	Ovarian serous adenocarcinoma	PubMed=22115851, 25877200	Kyoto University
OVCA433	-0.105				CVCL_0475	Ovarian serous adenocarcinoma	PubMed=25877200	Kyoto University
JHOS2	-0.100	✓		✓	CVCL_4647	High grade ovarian serous adenocarcinoma	RCB	RIKEN
OVCA429	-0.079			✓	CVCL_3936	Ovarian cystadenocarcinoma	PubMed=25877200	Kyoto University
CAOV3	-0.076	✓	✓	✓	CVCL_0201	High grade ovarian serous adenocarcinoma	ATCC	ATCC
OAW28	-0.075	✓	✓	✓	CVCL_1614	High grade ovarian serous adenocarcinoma	ECACC	ECACC
JHOS3	0.067			✓	CVCL_4648	Ovarian serous adenocarcinoma	Cosmic-CLP; RCB	RIKEN
OVCAR3	0.119	✓	✓	✓	CVCL_0465	High grade ovarian serous adenocarcinoma	ATCC	ATCC
UWB1.289	0.133		✓		CVCL_B079	Ovarian carcinoma	ATCC	ATCC
DOV13	0.139			✓	CVCL_6774	Ovarian adenocarcinoma	PubMed=25877200	M.D. Anderson Cancer Center
OVCA432	0.181				CVCL_3769	Ovarian serous adenocarcinoma	PubMed=25877200	Kyoto University
OV56	0.190	✓	✓		CVCL_2673	Ovarian serous adenocarcinoma	ECACC	ECACC
EFO21	0.191	✓		✓	CVCL_0029	Ovarian cystadenocarcinoma	DSMZ	DSMZ
OAW42	0.237	✓	✓	✓	CVCL_1615	Ovarian cystadenocarcinoma	ECACC	ECACC
FUOV1	0.271	✓		✓	CVCL_2047	High grade ovarian serous adenocarcinoma	DSMZ	DSMZ
OVCAR5	0.272			✓	CVCL_1628	High grade ovarian serous adenocarcinoma	PubMed=19372543, 25877200	NCI-Frederick
OV7	0.311		✓		CVCL_DG86	Ovarian serous cystadenocarcinoma	---	ECACC
OVCAR2	0.345				CVCL_3941	Ovarian carcinoma	---	Kyoto University
OV17R	0.362		✓		CVCL_2672	Ovarian adenocarcinoma	ECACC	ECACC
COLO720E	0.386		✓		CVCL_1995	High grade ovarian serous adenocarcinoma	PubMed=22710073	ECACC
SKOV3	0.403	✓	✓	✓	CVCL_0532	Ovarian serous cystadenocarcinoma	ECACC	ATCC
CH1	0.413				CVCL_4992	Ovarian carcinoma	PubMed=11416159	Kyoto University
TYKNU	0.434	✓		✓	CVCL_1776	High grade ovarian serous adenocarcinoma	JCRB; PubMed=25877200	JCRB
HEY	0.462			✓	CVCL_0297	High grade ovarian serous adenocarcinoma	PubMed=25877200	Kyoto University
HEYA8	0.470	✓			CVCL_8878	High grade ovarian serous adenocarcinoma	---	Kyoto University
HEYC2	0.472				CVCL_X009	High grade ovarian serous adenocarcinoma	---	Kyoto University
TOV112D	0.511		✓	✓	CVCL_3612	Ovarian endometrioid adenocarcinoma	ATCC	ATCC
A2780	0.532	✓	✓	✓	CVCL_0134	Ovarian endometrioid adenocarcinoma	ECACC	ECACC

Supplementary Table 2. Primers used for RT-qPCR (gene expression), ChIP-qPCR and methylation-specific PCR.

For gene expression (RT-qPCR):

ACTB
From Qiagen, catalog #: PPH00073E

GAPDH
From Qiagen, catalog #: PPH00150E

CDH1
From Qiagen, catalog #: PPH00135E

ZEB1
From Qiagen, catalog #: PPH01922A

ESRP1
From Qiagen, catalog #: PPH088556A

VIM
From Qiagen, catalog #: PPH00417E

CLDN4
From Qiagen, catalog #: PPH07330D

GRHL2

Forward:	5' CTCAGTATGACGTGCCCTCGCTG 3'
Reverse:	5' GGTGGCTTCCAGGGTGTACTGAA 3'

OVOL2

Forward:	5' GCCCAAAGTCTTCCTGGTGAA 3'
Reverse:	5' TAGGCCCACTGGGATGTAGGT 3'

PROM2

Forward:	5' GTGAATGAGGTGGTGCGGTA 3'
Reverse:	5' GCTCTGTCTTCACTCGTCCC 3'

CGN

Forward:	5' AGCCCGGCTAGGGACTC 3'
Reverse:	5' CCGTAGGTACTGGCTCTTGC 3'

PVRL4

Forward:	5' ACAACTGGACACGGCTGGAT 3'
Reverse:	5' CAGAGTCTTCCTGGGGGTCA 3'

S100A14

Forward:	5' TGTCCTCATCTCTGCAAAGTTCAGC 3'
Reverse:	5' TGGCCCAACCAGTCCCCTGAGCCTC 3'

SPINT1

Forward:	5' CACGTCCTGAGAAAGCTCAAAGGTT 3'
Reverse:	5' GGGACAGGCAGCTTTGAACAGAGGA 3'

For ChIP-qPCR:

CDH1 promoter

Forward:	5’ GGCCGGCAGGTGAACCCTCA 3’
Reverse:	5’ GGGCTGGAGTCTGAACTGA 3’

CDH1 intron 2

Forward:	5' TTCAAAAGATCCCCTGCGCT 3'
Reverse:	5' AAGCCACAACAAACCCGTTC 3'

GRHL2 promoter

Forward:	5’ AGCTCTTGTTCTGCCATCTCG 3’
Reverse:	5’ CCATCAGCCCAAAGTGGTAGT 3’

GRHL2 intron 8

Forward:	5’ GTGTAATGTGAACAGGCGTCC 3’
Reverse:	5’ CGAGTGCCAGGGCTAGAAT 3’

OVOL2 promoter

Forward:	5’ CCGCCCGGTGGATTTGTCTTC 3’
Reverse:	5’ GACCGCCCAGATTTCATCAAGC 3’

For methylation-specific PCR:

CDH1 MethylF:	5’ TAATTTTAGGTTAGAGGGTTATCGC 3’
CDH1 MethylR:	5’ CTCACAAATACTTTACAATTCCGAC 3’
CDH1 UnMethylF:	5’ ATTTTAGGTTAGAGGGTTATTGTGT 3’
CDH1 UnMethylR:	5’ CAAACTCACAAATACTTTACAATTCCA 3’

Supplementary References

1. Liberzon, A. et al. The Molecular Signatures Database (MSigDB) hallmark gene set collection. *Cell systems.* **1**, 417-425 (2015).

2. The Gene Ontology Consortium The Gene Ontology Resource: 20 years and still GOing strong. *Nucleic Acids Res.* **47**, D330-D338 (2019).

3. The Cancer Genome Atlas Program. *National Cancer Institute* https://www.cancer.gov/tcga

4. Yamaguchi, K. et al. Epigenetic determinants of ovarian clear cell carcinoma biology. *Int J Cancer.* **135**, 585-597 (2014).

5. Wu, X. et al. Clinical importance and therapeutic implication of E-cadherin gene methylation in human ovarian cancer. *Med Oncol.* **31**, 100 (2014).
