# Supplementary material for: The role of GRHL2 and epigenetic remodeling in epithelial–mesenchymal plasticity in ovarian cancer cells
Source: Commun Biol. 2019 Jul 24;2:272. doi: 10.1038/s42003-019-0506-3 (PMC6656769; doi:10.1038/s42003-019-0506-3)
Supplement: Supplementary file 4 — Description of Additional Supplementary Items [file 42003_2019_506_MOESM4_ESM.docx]

**Supplementary Data 1. EMT-correlated DMCs.**

Differentially methylated CpG sites (DMCs) identified by the probes of Infinium HumanMethylation450K BeadChip array are listed along with the associated genes, regulatory elements, CpG islands, the correlation of CpG methylation with the EMT score (*Rho* and *p* values; EMT+ or EMT−), and the association of these DMCs with ovarian cancer EMT signature genes and DNA-binding sites (1 = yes; 0 = no). EMT refers to epithelial-mesenchymal transition.

**Supplementary Data 2. EMT-correlated DMCs cross-referenced with gene expression.**

Differentially methylated CpG sites (DMCs) identified by the probes of Infinium HumanMethylation450K BeadChip array are listed along with the associated genes, regulatory elements, CpG islands, the correlation of CpG methylation with the EMT score (*Rho* and *p* values), the correlation of EMT score with the expression of associated genes (*Rho* and *p* values), and the overlap with ovarian cancer EMT signature (−1 = Mes gene; +1 = Epi gene; 0 = no overlap).

**Supplementary Data 3. Ovarian cancer EMT signature genes with EMT-correlated DMCs at different genomic regions.**

EMT signature genes (Epi or Mes genes) and the respective number of EMT+ DMCs and/or EMT− DMCs located at promoter regions or in gene bodies. TSS = transcription start site; UTR = untranslated region.

**Supplementary Data 4. EMT-correlated DMCs in different EMT signature gene sets.**

A list of hallmark EMT genes from Msigdb v6.1 gene set and epithelial differentiation genes from Gene Ontology gene set (with or without overlap with ovarian cancer EMT signature genes) that harbor DMCs at TSS or in gene bodies. The correlation of EMT score with gene expression (*Rho* and *p* values) and the correlation of CpG methylation (TSS/gene body) with the EMT score (*Rho* and *p* values) are shown alongside. '---' or NaN indicates no data. TSS = transcription start site.

**Supplementary Data 5. Enrichment of EMT-correlated DMCs in ChIP-seq database ReMap 2018 v1.2.**

A list of DNA-binding factors with DNA-binding sites (based on ChIP-seq database ReMap 2018 v1.2) that overlap with EMT-correlated DMCs identified in this study. The number of DMCs (in all region or in promoter) that overlap with the respective binding sites, the –log(E-value) and the EMT correlation of the DMCs (EMT+ or EMT−) are shown alongside. Grey-highlighted cells indicate no significant enrichment/overlap. NaN indicates no overlap of peaks.

**Supplementary Data 6. DMCs with differential CpG methylation and associated gene expression in the GRHL2-knockdown model.**

DMCs (CpG probes) with the associated genes, regulatory elements, CpG islands, overlap with GRHL2 binding sites, the correlation of CpG methylation with the EMT score (*Rho* and *p* values) are shown alongside their respective differential methylation (∆β-values and *p*-values of two-tailed unpaired *t*-tests) in OVCA429 shGRHL2 vs. control cells. The differential expression of the genes associated with these DMCs in OVCA429 shGRHL2 vs. control cells is also shown (fold change and PPDE calculated from EBseq). PPDE = posterior probability of differentially expressed. '---' or NaN indicates no data.

**Supplementary Data 7. ChromHMM state changes (at promoters) of EMT signature genes clustered based on DNA methylation and histone modifications.**

Ovarian cancer EMT signature genes (Epi/Mes) that are subdivided into 6 clusters (A to F) by unsupervised hierarchical clustering are listed with details of the promoters (transcript, chromosome, promoter start site, promoter end site) and the ChromHMM state changes in both the four-cell-line model and the GRHL2-knockdown model. The differential expression of these genes in OVCA429 shGRHL2 vs. control cells is shown alongside (fold change and PPDE calculated from EBseq). PPDE = posterior probability of differentially expressed.

**Supplementary Data 8. ChromHMM states of GRHL2 binding sites in the GRHL2-knockdown model.**

GRHL2 binding sites at different chromosomes are listed along with their loci (start and end), genomic annotations, associated genes, respective dominant ChromHMM states in OVCA429 control and shGRHL2 cells and the differential expression of associated genes in OVCA429 shGRHL2 vs. control cells (fold change and PPDE calculated from EBseq). PPDE = posterior probability of differentially expressed.

**Supplementary Data 9. Co-occupancy analysis of GRHL2 and other DNA-binding factors.**

DNA-binding factors with ChIP-seq data available are listed by their ranks in co-occupancy with GRHL2 (overlap of their respective binding sites with GRHL2 binding sites). The functional annotations of the factors (curated on EpiFactor database) and the average -log10(E-value) are listed alongside.

**Supplementary Data 10. SILAC-co-IP-mass spectrometry analyses using anti-GRHL2 antibody performed in PEO1 and OVCA429 cells.**

Proteins identified from SILAC (Stable Isotope Labeling with Amino acids in Cell culture)-coupled co-IP (co-immunoprecipitaton) followed by mass spectrometry are listed by their ranks in normalized Heavy/Light (H/L) ratio in GRHL2 pulldown vs. IgG control. The analyses were performed in PEO1 (top panel) and OVCA429 (bottom panel) cells.
